# A grey wolf optimization-based modified SPWM control scheme for a three-phase half bridge cascaded multilevel inverter

**DOI:** 10.1038/s41598-024-57262-0

**Published:** 2024-03-25

**Authors:** Abdelrahman M. Nasser, Amr Refky, Hamdy Shatla, Alaa M. Abdel-hamed

**Affiliations:** 1 Department of Electrical Engineering, Faculty of Engineering, Al-Azhar University, Cairo, Egypt; 2 Electrical Power and Machines Department, High Institute of Engineering, El-Shorouk Academy, Cairo, Egypt

**Keywords:** Multilevel inverter (MLI), Renewable energy (RE), Modified sinusoidal pulse width modulation (SPWM), Grey wolf optimization (GWO), THD, GA, PSO, Engineering, Electrical and electronic engineering

## Abstract

The Multilevel inverter (MLI) plays a pivotal role in Renewable Energy (RE) systems by offering a cost-effective and highly efficient solution for converting DC from Photovoltaic (PV) sources into AC at high voltages. In addition, an innovative technology holds immense significance as it not only enables the seamless integration of PV systems into the grid but also ensures optimal power generation, thereby contributing to the widespread adoption of RE and fostering a sustainable future. This paper presents a modified sinusoidal pulse width modulation (SPWM) control scheme for a three-phase half-bridge cascaded MLI-powered PV sources. The selection of the MLI configuration is motivated by its reduced number of switching components, which enhances system reliability and simplifies experimental implementation. Compared to the SPWM schemes which require (m−1) carriers that make the generation of the pulse circuit very complex, the proposed control scheme requires only three signals: a carrier signal, a triangular waveform, and a modulating signal. This approach significantly reduces the complexity of control and facilitates practical implementation. The proposed control scheme simulation is verified using MATLAB/SIMULINK Software. The grey wolf optimization (GWO) algorithm is implemented to determine the optimal switching angles of the proposed control scheme. The Total Harmonic Distortion (THD) objective is selected to be the fitness function to be minimized for improving the quality of the output waveforms. For verification, the results of the proposed GWO-based modified SPWM control scheme are compared with those obtained using both the Particle swarm Optimization (PSO) and Genetic algorithm (GA) used in the literature. Simulation results declared that the proposed control scheme improves performance, especially THD which is minimized to 6.8%. Experimental validation has been conducted by building a laboratory prototype of the proposed system. The experimental and simulation results gave acceptable and limited convergent results considering the experimental difficulties.

## Introduction

The rapid adoption of Renewable Energy Sources (RESs) in Low-Voltage (LV) networks has extremely motivated considerable research interest in developing more efficient, compact, dependable, and easily expandable energy conversion devices^1^. Among the various concepts of DC-AC conversion, Multi-Level Inverters (MLIs) have emerged as a promising solution^2^. These converters are gaining popularity due to their smaller size and voltage ratings for controlled switches when compared to conventional two-level structures, but the number of switches should be higher. Particularly, MLIs have garnered significant attention due to their small size, high reliability, and remarkable efficiency^3^. These qualities make them highly useful across various applications, from LV scenarios to high-power, High-Voltage (HV) converters employed in Photovoltaic (PV) and wind power systems^4,5^. The attractiveness of MLIs lies in their ability to provide enhanced performance and improved energy conversion capabilities, contributing to the ongoing advancements and wider implementation of RE technologies in LV networks^6^.

These multilevel topologies are commonly divided into three major groups: Neutral-Point Clamped (NPC), Flying Capacitor (FC), and cascaded, which are further subdivided into Modular Multilevel Converter (MMC) and Cascaded H-Bridge (CHB)^7,8^. New topologies have been developed to overcome the associated drawbacks of traditional MLIs. The new MLI topologies take two approaches: one reduces the number of switching components, and the other reduces component power rating^9^.

The main utilization of MLI can be classified into two distinct groups. The first is employed in large-scale motor drives which encompass various industrial sectors such as the petrochemical industry, the cement industry, and the transportation industry, besides in the utilization of blowers, compressors, conveyors, and downhill conveyor systems. The second MLI finds applications in power systems, especially in devices such as STATCOM, Unified Power Flow Control (UPFC), power quality improvement, power conditioners, reactive power compensators, and grid-connected systems. Additionally, MLI has been reported to be efficiently utilized in rectifiers, DC-to-DC converters, fuel cell utilization, and arc furnaces^10^.

Despite the advantages of integrating MLIs with RES in the electrical power system, the existence of harmonics reduces the performance and efficiency of MLIs. Numerous research studies have been introduced since the latest decades to mitigate the harmonics content. Based on various research, the Selective-Harmonic Elimination-based Pulse Width Modulation (SHE-PWM) method has been verified to be the best choice for minimizing the low order harmonics. However, when deterministic-based methods are implemented to solve the nonlinear equations and systems, these methods have shown some complications. Application of iterative-based techniques to solve the SHE-PWM optimization problems, such as the Newton–Raphson (NR) technique can get stuck in local optima. Additionally, these techniques are sensitive to the initial values calculation of the required solution. In contrast, Artificial Intelligence (AI) methods perform to be a better solution to these problems^11,12^.

Rao, S.N.V.B, et al. in^13^ introduced a renewable-based microgrid cluster by integrating several buildings implemented in urban communities. The available energy of the microgrid cluster is managed to enhance the power supply reliability. A fuzzy space vector PWM methodology is proposed for controlling the implemented inverter, which increases the power quality of the supply. The Fuzzy Logic (FL) is used to optimize the reference currents, which were implemented for plotting the space vectors by effectively selecting the sector for generating the precise PWM-controlled signals of the inverter. The efficacy of the introduced method was proved by plotting various characteristics. The results indicated that the proposed inverter adhered to entirely standard requirements. The authors in^14^ proposed a novel control method “adaptive neuro-fuzzy control strategy” to control the implemented inverter for each microgrid and the converter-based compensator located between the utility grid and cluster. This proposed strategy implements the advantages of both neural networks and FL. Several power quality indices such as THD and voltage unbalance are measured. The superiority of the introduced controller is proved by comparison with Conventional and FL-based controllers about the key IEEE/IEC standards.

Kumar et al. in^15^ proposed asymmetric MLI that can be used for PV uses. This topology of the MLI implements a smaller number of switches and DC sources. The proposed topology is controlled by implementing SHE-PWM to eliminate the lower-order harmonics. Particle Swarm Optimization (PSO) implemented with the Newton–Raphson approach is used to solve the nonlinear equalities formed by the SHE-PWM and obtain the proper switching angles for the proposed topology of the MLI. The effectiveness of the proposed topology is investigated using the THD for various operating levels and comparing similar approaches in the literature.

Kenia Díaz et al. in^16^ presented the minimization of the THD in a seven-level MLI with resistive load by implementing the Teaching–Learning-based Optimization (TLO) algorithm. The obtained results using the suggested technique were compared with the Genetic Algorithm (GA), PSO, and differential evolution optimization algorithm. TLO algorithm presented an acceptable THD reduction, Furthermore, the control parameters operation is more convenient.

Injila Sajid et al. in^17^ implemented the Runge Kutta Optimization (RKO) algorithm to validate the SHE-PWM method in several topologies of MLIs. Various Modulation Indices (MIs) are used to obtain the switching angles. The advantage of the implemented method is verified by comparing the obtained results with those obtained using the GA, Differential Evolution (DE), and GWO methods. The switching angles found using RKO algorithm are experimentally verified to give better performance. The RKO method has better performance than the other methods which were used for comparison.

Boucheritte et al. in^18^ introduced a grid-connected PV shared feeding an MLI. A 5-level neutral point topology is used for integrating the PV with the grid at minimum THD and high capacity of power. The ripple of the output voltage is fixed with the variation of the output voltage resulting from the variation of the solar radiation. GA optimization is implemented to tune the parameters of the PI controller which was used as a regulation loop in the proposed topology. The system was simulated, and the simulation results demonstrated the stability and accuracy of the proposed topology.

Davari et al. in^19^ proposed a new topology of asymmetrical-type MLI with a minimized component count. The number of active switches is minimized to three in the conduction track. A new solution using the PSO algorithm is proposed to tune the magnitude of the DC sources. The optimal switching angle technique is implemented to find the most suitable angles using the PSO algorithm. The effectiveness of the proposed approach is verified by comparing the results with those obtained using conventional MLI. Validation, implementing a single-phase prototype, is performed.

Siddique et al. in^20^ introduced a novel single-phase cascaded-type MLI topology. The suggested topology is designed to reduce both the number of DC sources and switches, while having a larger number of levels. Three different optimization techniques are implemented to determine the number of levels and magnitude of voltage sources while optimizing the other parameters. SHE-PWM is implemented to determine the pulses used to achieve high-quality output voltage. Experimental results are performed to validate the proposed topology.

Uthirasamy et al. in^21^ proposed a novel fifteen-level Hybrid converter for solar-type PV system applications. A boost-type chopper is designed with converters and implemented in brushless DC drive systems. The PV output is stepped up and regulated to achieve the nominal voltage. The DC-link modules are used to obtain DC-link voltage for the fifteen-level with minimized electrical energy compression. The introduced topology achieved minimized power loss and switching. In comparison with other converters, the proposed topology is more reliable due to the reduction of gate firing circuits and power semiconductor switches. Experimental results showed a reduction of 54% in controlled switches and 7% reduction in harmonics.

Xinxiao Qin, et al. in^22^ presented a PSO algorithm with a modulation method to obtain improved SHE-PWM switching angles. The switching angles are optimized to reduce the THD and the output voltage error. The proposed methodology is implemented to improve the waveform quality of the current. The PSO is implemented to optimize the joint function to find the optimal switching. The suggested method is verified via simulation results indicating its ability to obtain the switching angles with minimized THD and voltage error.

Srilakshmi et al. in^23^ introduced a hybrid active-power filter integrated with wind, solar, and energy storage systems. A three-level converter of shunt type is employed and linked to the DC bus. The gains of the fractional order-type PID controller and the parameter values of the implemented filter are optimized by implementing a jaya grey wolf-hybrid technique. The performance of the proposed controller is achieved using various test setups of different loads and irradiation levels. The GA, PSO, and ant colony optimization techniques are implemented to design the proposed controller and to assess their respective contributions in the improvement of the results.

This paper presents a detailed description of a three-phase MLI. It is designed with ten switches and three distinct DC sources, enabling the generation of a fifteen-level output voltage. Furthermore, the paper discusses and analyzes a modified SPWM technique employed for controlling the inverter. A Grey Wolf Optimization (GWO) technique is subsequently applied to determine the optimal inverter switching angles, aiming to minimize THD and compare the performance of the inverter with other optimization techniques used in the literature. The proposed control scheme is emphasized for its efficiency and simplicity, requiring only three signals to achieve the required pulses. Additionally, the importance of optimization techniques implemented in the model is discussed, aiming to obtain better results and minimize THD. As a result, the output voltage waveform approximates a pure sine wave, eliminating the need for additional filtering requirements.

The primary contributions of this paper can be summarized as follows:

(i) A comprehensive model is developed for a three-phase 15-level cascaded half bridge MLI.
(ii) A modified SPWM control scheme for a three-phase half-bridge cascaded MLI powered by photovoltaic sources is proposed.
(iii) The GWO technique is implemented to find the optimal switching angles that effectively minimize THD.
(iv) A comparative analysis is conducted to evaluate the performance of the inverter in relation to other optimization techniques, such as GA and PSO. The choice to use (GA) and (PSO) since they are well-known and widely used optimization techniques with a proven track record of success in power electronics and by comparing the performance of the inverter against these established methods, researchers can provide a reference point for evaluating the effectiveness and efficiency of their proposed optimization approach.
(v) Experimental validation of the proposed MLI system.

The remaining sections of the paper are structured as follows:

The “Complete system modeling” section provides an in-depth discussion of MLI definition, classification, advantages, problems, and system modeling with PV. The “Modified SPWM technique for MLI” section introduces the SPWM control method and its relevance to the MLI system. The “GWO technique” section describes the implemented GWO technique. The “Proposed control objective and optimal-tuning implementation” section implements the GWO technique and compares it with GA and PSO techniques. The “Simulation results” section presents simulation results for various study cases, analyzing the performance of the MLI system with SPWM control and GWO. The “Experimental validation” section demonstrates experimental validation of the proposed MLI system. Finally, the paper concludes in “Conclusion” section, summarizing the overall findings and potential research directions.

## Complete system modeling

**Figure 1:**
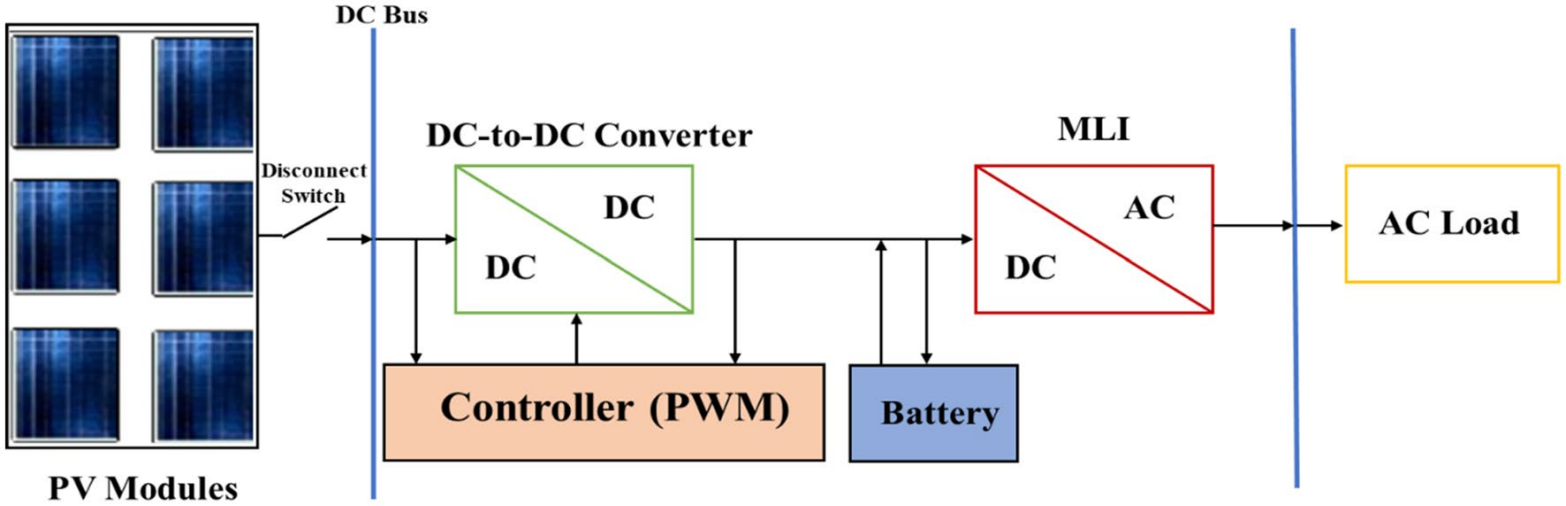
Block diagram of a stand-alone PV system supplied by MLI.

In the following sub-section, the mathematical model of the complete system shown in Fig. 1 is investigated.

### Solar PV systems modeling

**Figure 2:**
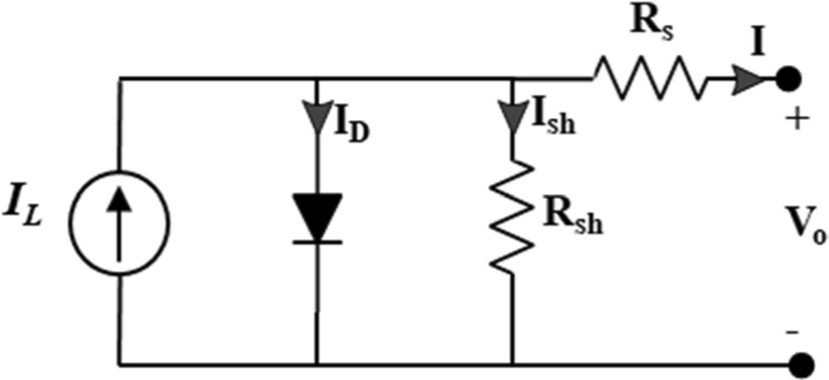
Single diode model of the PV system.

PV systems integrated with MLIs offer advantages for efficient power conversion and grid integration^24–26^, harnessing solar radiation and converting it into AC power suitable for grid or stand-alone loads^27^. The integration provides a reliable solution for RE conversion^28^. The PV system, represented by the five-parameter single-diode model, is shown in Fig. 2. The model describes the current–voltage characteristics using a nonlinear Eq. (1), involving parameters such as light current, reverse saturation current, series resistance, shunt resistance, and diode ideality factor^29^. An Inventux X3-125 PV model is used in this paper. The implemented module is specifically micro-morph type (a-Si/µc-Si). Its datasheet can be found in Online Appendix A.

1 $$I={I}_{L}-{I}_{o}[{e}^{(\frac{V+I.{R}_{s}}{n.{V}_{T}})-1}]-\frac{V+I.{R}_{s}}{{R}_{sh}}$$

where ${I}_{L}$ is the light current, ${I}_{o}$ is the diode reverse saturation current, ${R}_{s}$ is the series resistance, ${R}_{sh}$ is the shunt resistance, and ${\text{n}}$ is the diode ideality factor.

### Three-phase half-bridge cascaded MLI

This section outlines the rationale behind selecting the CHB-MLI, draws comparisons with traditional MLIs, and engages in a detailed examination and evaluation of the CHB-MLI model.

### Comparative analysis with traditional inverters

In contrast to traditional inverters that rely on a single-voltage level, the MLI achieves voltage synthesis by combining multiple levels of DC voltages, generating higher voltage levels, and reaching several kilovolts^30^. This attribute proves advantageous in applications necessitating high-voltage power transmission or integration with HV systems^31^.

The MLI offers several notable advantages, including its ability to operate at lower switching frequencies compared to conventional inverters^32^. Consequently, it exhibits reduced switching losses, resulting in enhanced energy efficiency. Furthermore, the output waveforms of the MLI exhibit a stepped configuration, leading to diminished harmonic content and lower rates of voltage change $\frac{dV}{dt}$ when compared to square wave or PWM inverters^33^. Consequently, the MLI proved to be well-suited for applications that prioritize low harmonic distortion and minimize voltage stress on connected devices^34^.

There exist three primary classifications of MLI: NPC, FC, and CHB^35^. The FC inverters present challenges in their practical implementation due to the requirement of varying voltage charging for each capacitor as the voltage level increases^36^. Similarly, the expansion of NPC inverters into multilevel configurations is complicated by inherent issues such as DC link voltage imbalance, the escalation in the number of clamping diodes, and the intricate arrangement of DC link capacitors and devices as voltage levels rise^37^. Conversely, although CHB inverters necessitate separate DC sources, they offer the potential for modularized circuit layout and packaging, while circumventing concerns regarding DC link voltage imbalance. Consequently, the CHB inverter was selected for utilization in this study, owing to its advantageous characteristics^38^.

### Control techniques of MLIs

**Figure 3:**
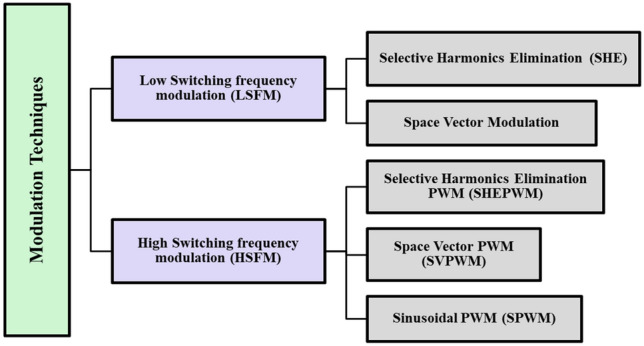
MLI Control- classifications.

Different modulation techniques can be used to control MLIs ^39^. These techniques are classified based on their switching frequency to the Fundamental Switching Frequency Modulation (FSFM) and the High Switching Frequency Modulation (HSFM)^40^. Both methods produce a stepped output waveform. However, at high-switching frequency methods, steppes are modulated with PWM^41^. Figure 3 categorizes and depicts the various MLI control techniques.

### Half bridge cascaded MLI concept

**Figure 4:**
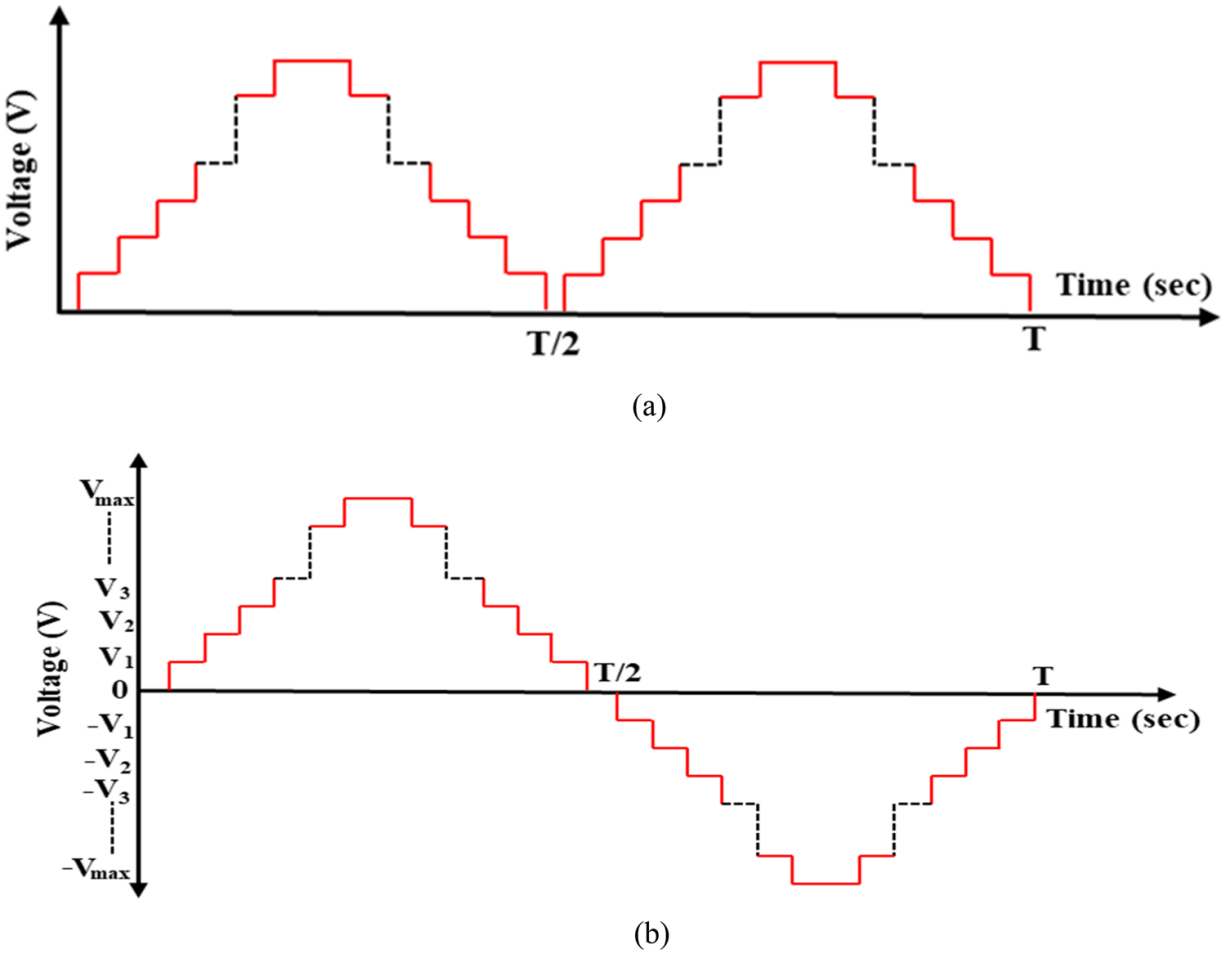
Output voltage of CHB- MLI.

The adopted MLI comprises two stages: the primary stage and the polarity stage. This MLI can produce greater numbers of levels using smaller numbers of switches, albeit with higher power ratings. Following the polarity stage, the inverter generates the output voltage^42^. The primary stage consists of multiple fundamental units, each of which incorporates only two series-connected switches. These units can generate solely two specific voltage levels: either $0$ or ${V}_{dc}$, with each unit possessing its own dedicated DC voltage source. The primary stage’s fundamental function is to generate the waveform levels of the cascaded units. To avoid short circuits across the DC source, the two switches (${S}_{1}$ and ${S}_{2}$) in each unit are alternately activated, ensuring their complementary operation^40^. The voltage produced by each unit is subsequently combined, resulting in a cumulative voltage that is either zero or a positive value after the primary stage as shown in Fig. 4a. The polarity stage, resembling a traditional H-bridge configuration, is employed to convert the voltage generated by the primary stage into AC voltage. During the initial half-cycle, the polarity of the voltage aligns with that of the primary stage, whereas during the subsequent half-cycle, the polarity is reversed as depicted in Fig. 4b, effectively transforming the DC voltage into AC voltage. This configuration enables the generation of multiple voltage levels using a reduced number of switches, rendering it a viable choice for various applications, including motor drives and RE systems.

When the fundamental units are supplied by DC sources of equal magnitudes, the levels are determined using Eq. (2). For instance, if $n$ equals three, the corresponding levels would be seven. The number of levels can be increased without adding more fundamental units if the primary stage is powered by DC sources of different magnitudes. By adjusting the voltage magnitudes of each unit in the primary stage, a different number of levels can be achieved.

The maximum value of the controllable level of the DC voltage is determined by the sum of individual voltage magnitudes, denoted as ${v}_{{o}_{1}},{v}_{{o}_{2}},{v}_{{o}_{3}}$, and so on, up to ${v}_{{o}_{n}}$. It can be expressed as Eq. (3) indicates.

2 $$m=2n+1$$

3 $${V}_{d{c}_{out}}={v}_{{o}_{1}}+{v}_{{o}_{2}}+{v}_{{o}_{3}}+.....+{v}_{{o}_{n}}$$

The determination of the number of voltage levels ($m$) depends on the configuration and magnitudes of the DC supplies. The commonly employed formula indicated by Eq. (2) calculates the number of voltage levels when all cascaded fundamental units possess equal DC supplies. For instance, if three cascaded fundamental units are employed, implementing Eq. (2) results in seven voltage levels. Nevertheless, it is feasible to achieve the same number of voltage levels with only two cascaded fundamental units by utilizing distinct DC supplies. This is where the symmetric, binary, trinary, and duplicated methods come into play. The symmetric method involves employing DC sources of equal magnitudes, while still adhering to the formula indicated in Eq. (2) ^43^. In the binary method, the magnitudes of the DC sources are determined through an exponential progression of a binary factor. The i^th^ DC source magnitude is calculated using the formula indicated by Eq. (4). This method allows for the attainment of the desired number of voltage levels with a reduced number of cascaded fundamental units.

4 $${V}_{i}=({2}^{i-1}){V}_{dc}$$

where ${V}_{dc}$ represents a constant denoting the base magnitude of the DC supply.

Similarly, the trinary method determines the magnitudes of the DC sources through an exponential progression of a trinary factor. The formula indicated by Eq. (5) calculates the magnitude of the ith DC source. Like the binary method, this approach achieves the desired number of voltage levels with fewer cascaded fundamental units.

5 $${V}_{i}=({3}^{i-1}){V}_{dc}$$

The duplicated technique retains the initial DC source value while duplicating the remaining DC sources. By using this method, the desired number of voltage levels can be achieved with a reduced number of cascaded fundamental units. It is crucial to consider that the selection of the method depends on the specific requirements and limitations of the inverter system. Each method possesses its advantages and limitations, and the appropriate approach should be chosen based on the desired number of voltage levels, available resources, and system constraints. Based on the analysis presented in Table 1, it is evident that the asymmetric (binary) method offers a notable enhancement in the number of voltage levels achievable for employing the same number of units. Conversely, the trinary method proves inappropriate for these specific types of inverters, as it fails to generate certain combinations of DC sources required to achieve the desired voltage levels.

**Table 1:** Comparison of four methods of feeding the MLI.

Configuration	${V}_{o max}$	Number of steps $({N}_{step})$	Number of levels $(m)$	$m if (n=3)$	Percentage reduction in the number of switches
Symmetric	$n.{V}_{dc}$	$n+1$	$2n+1$	7	$\left[1-\frac{m+3}{2\left(m-1\right)}\right]*100$
Duplicated	$2(n-1).{V}_{dc}$	$2n$	$4n-1$	11	$\left[1-\frac{\left({\text{m}}+9\right)}{4\left(m-1\right)}\right]*100$
Binary	${(2}^{n-1}).{V}_{dc}$	${2}^{n}$	$\left({2}^{n+1}\right)-1$	15	$\left[1-\frac{ln\left(2m+2\right)}{ln\left(2\right).\left(m-1\right)}\right]*100$
Trinary	$({3}^{n}-1/2).{V}_{dc}$	$({3}^{n}-1/2)$	${3}^{n}$	27	$\left[1-\frac{{\text{ln}}{(m-3)}^{2}+4}{2\left(m-1\right)}\right]*100$

**Figure 5:**
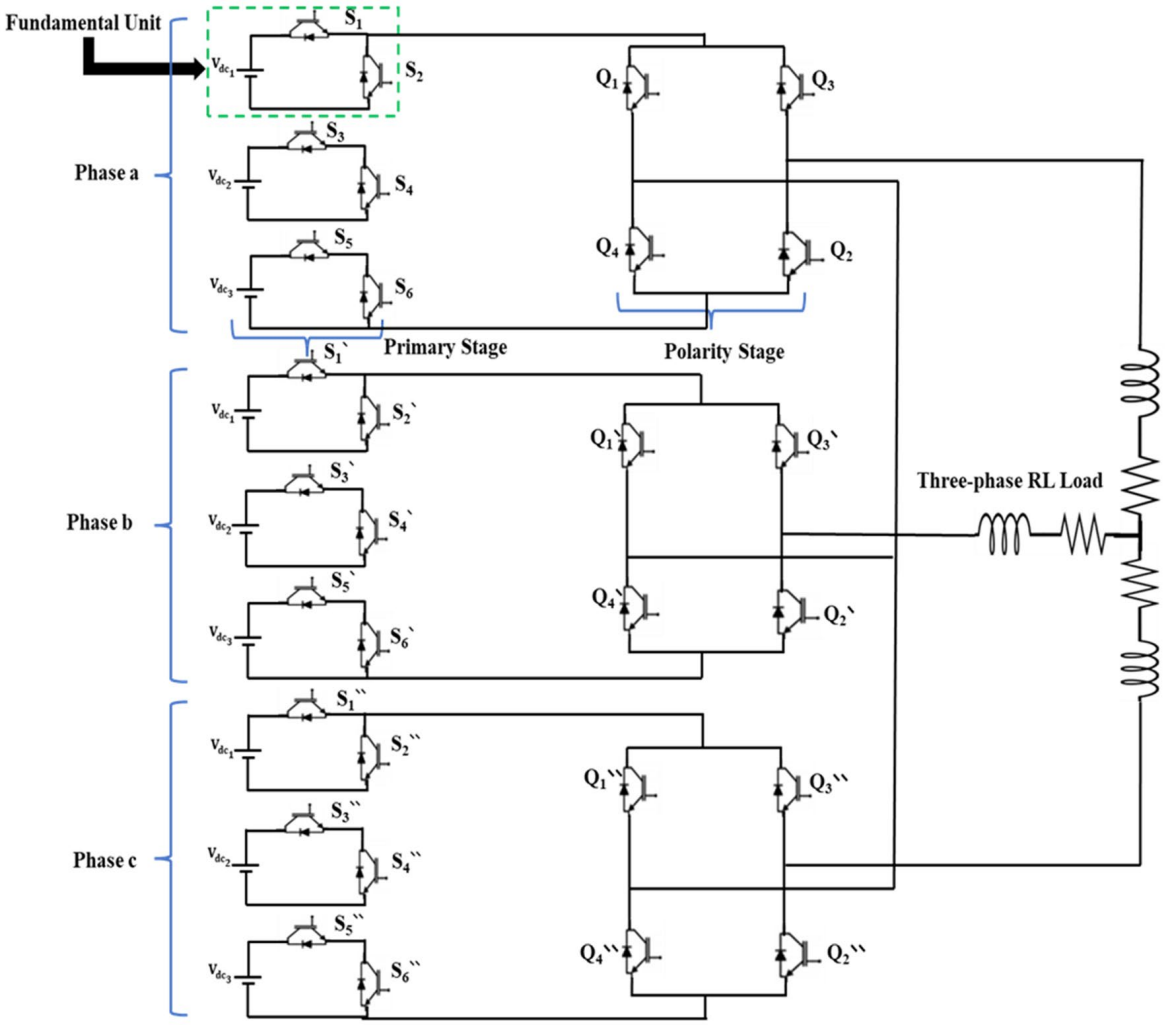
Circuit configuration of a three-phase 15-level MLI fed a RL load.

The three-phase 15-level MLI system comprises three identical phases. Each phase consists of ten switches, with six main switches functioning at a high frequency and four H-bridge switches controlled at a lower frequency. The inverter operates using a binary method to achieve fifteen levels of output. The circuit diagram for the implemented MLI model is shown in Fig. 5. Table 2 presents the corresponding switching states used to control the MLI system.

**Table 2:** Switching states pattern for the 15-level inverter used.

Output voltage (${V}_{out}$)			Primary stage						Polarity stage			
			${S}_{1}$	${S}_{2}$	${S}_{3}$	${S}_{4}$	${S}_{5}$	${S}_{6}$	${Q}_{1}$	${Q}_{2}$	${Q}_{3}$	${Q}_{4}$
Level 1	$0$	$0$	0	1	0	1	0	1	1	1	0	0
Level 2	${V}_{1}$	${V}_{dc}$	1	0	0	1	0	1	1	1	0	0
Level 3	${V}_{2}$	$2{V}_{dc}$	0	1	1	0	0	1	1	1	0	0
Level 4	${V}_{1}+{V}_{2}$	$3{V}_{dc}$	1	0	1	0	0	1	1	1	0	0
Level 5	${V}_{3}$	$4{V}_{dc}$	0	1	0	1	1	0	1	1	0	0
Level 6	${V}_{1}+{V}_{3}$	$5{V}_{dc}$	1	0	0	1	1	0	1	1	0	0
Level 7	${V}_{2}+{V}_{3}$	$6{V}_{dc}$	0	1	1	0	1	0	1	1	0	0
Level 8	${V}_{1}+{V}_{2}+{V}_{3}$	$7{V}_{dc}$	1	0	1	0	1	0	1	1	0	0
Level 9	$-{V}_{1}$	$-{V}_{dc}$	1	0	0	1	0	1	0	0	1	1
Level 10	$-{V}_{2}$	$-2{V}_{dc}$	0	1	1	0	0	1	0	0	1	1
Level 11	$-[{V}_{1}+{V}_{2}]$	$-3{V}_{dc}$	1	0	1	0	0	1	0	0	1	1
Level 12	$-{V}_{3}$	$-4{V}_{dc}$	0	1	0	1	1	0	0	0	1	1
Level 13	$-[{V}_{1}+{V}_{3}]$	$-5{V}_{dc}$	1	0	0	1	1	0	0	0	1	1
Level 14	$-[{V}_{2}+{V}_{3}]$	$-6{V}_{dc}$	0	1	1	0	1	0	0	0	1	1
Level 15	$-[{V}_{1}+{V}_{2}+{V}_{3}]$	$-7{V}_{dc}$	1	0	1	0	1	0	0	0	1	1
Number of switching			8	7	8	7	8	7	8	8	7	7

In a three-phase system, it is possible to interconnect three single-phase half-cascaded inverters with identical structures in either a star or delta configuration. According to the principles of three-phase theory, the line voltages can be expressed based on the phase voltages. Specifically, the voltage between phases $a$ and $b$, denoted as ${V}_{ab}$, can be calculated using Eq. (6), which states that it equals the difference between the phase voltages ${V}_{an}$ and ${V}_{bn}$. The maximum number of levels for line voltage is determined using Eq. (7). The three-phase systems can eliminate all triplen harmonic components in the line voltage.

6 $${V}_{ab}= {V}_{an}-{V}_{bn}$$

7 $${m}_{line}=2m-1$$

where *m* represents the number of levels,${V}_{ab}$ is the line voltage, ${V}_{an}$ , ${V}_{bn}$ are the phase voltage of phases A and B, respectively.

The choice of the 15-level MLI varies depending on the application and system requirements. However, there are a few general reasons such as:

(i) Improved output voltage quality: Higher-level MLIs, such as the 15-level inverter, can better approximate a sinusoidal output waveform than lower-level inverters. This improved output voltage quality can be desirable in applications that require low harmonic distortion and high-quality power output.
(ii) Reduced voltage stress: Increasing the levels number in the inverter reduces the voltage stress on each switching device. This reduction in voltage stress leads to improved reliability and longevity of the inverter components.
(iii) Higher power handling capacity: MLI with more levels generally have a higher power handling capacity. It is implemented in applications that require higher power ratings, such as grid-connected renewable energy systems or high-power motor drives.
(iv) Reduced switching frequency: In some cases, using a higher-level MLI can allow for a lower switching frequency. Lower switching frequency can help reduce switching losses and improve efficiency.

Selecting the 15-level MLI configuration offers the advantage of achieving a high-level number using a relatively small number of switches and three DC sources, such as PV panels. This configuration helps in reducing the physical size of the inverter and the overall cost associated with it. By effectively utilizing the available switches and DC sources, this approach provides a compact and cost-effective solution for achieving a higher level in the MLI system.

### Modified SPWM technique for MLI

The proposed technique is a High-Frequency Switching Modulation (HSFM) method based on SPWM. Its objective is to simplify the pulse generation process for the switches in the MLI system. In this proposed technique, only one carrier signal is used. This carrier is compared with the modulating signal. Additionally, a triangle wave is required, with the same frequency as the modulating signal. The modulating signal is a rectified sine wave with a magnitude (${A}_{m}$) and frequency (${f}_{m}$), while the carrier signal is a triangle wave with a magnitude (${A}_{c}$) and frequency (${f}_{c}$). The modulation index (${m}_{a}$) is the ratio between ${A}_{m}$ and ${A}_{c}$, and the modulation frequency (${m}_{f}$) is the ratio between ${f}_{c}$ and ${f}_{m}$.

**Figure 6:**
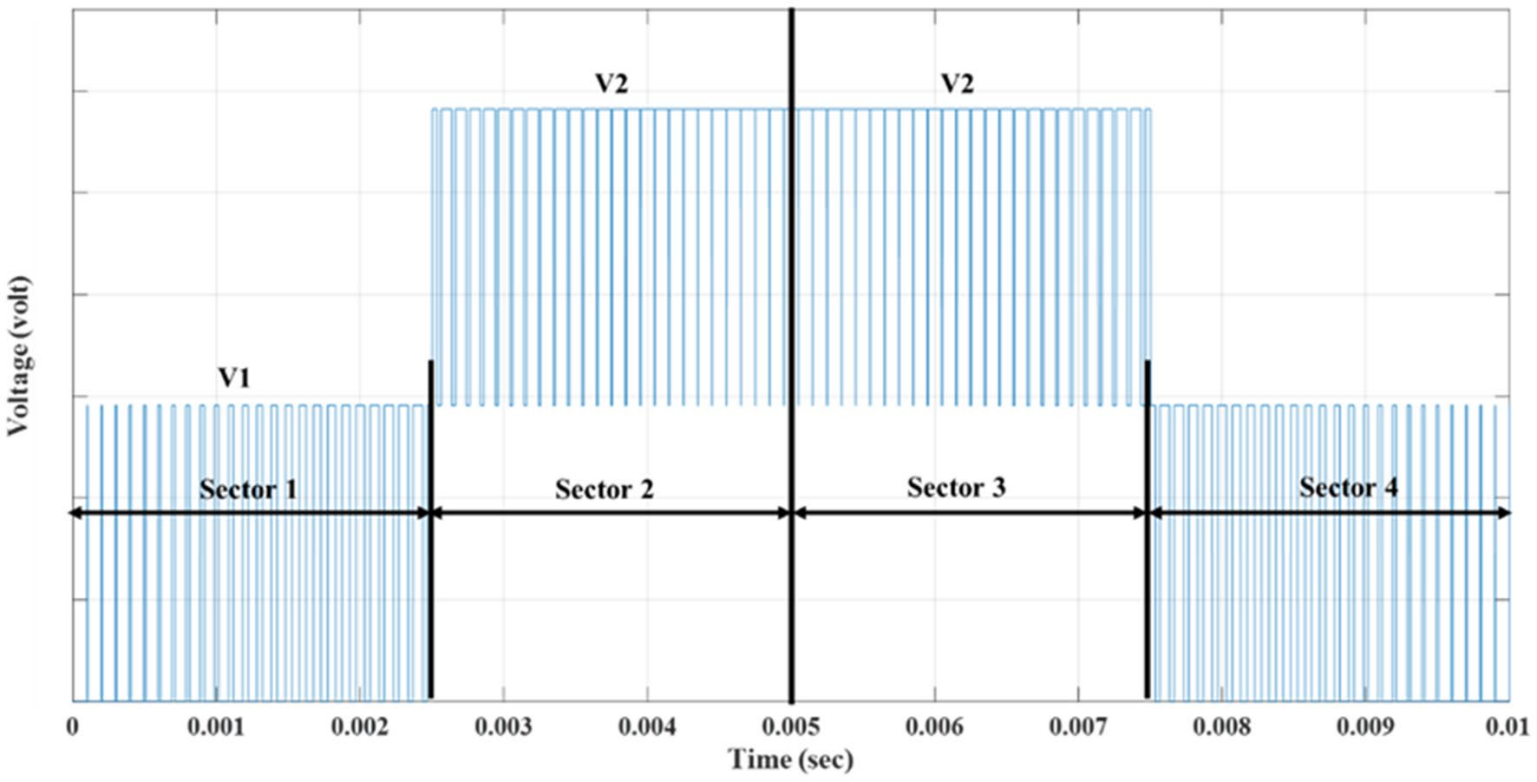
Inverter output voltage half-cycle waveform for a 5-level inverter.

**Figure 7:**
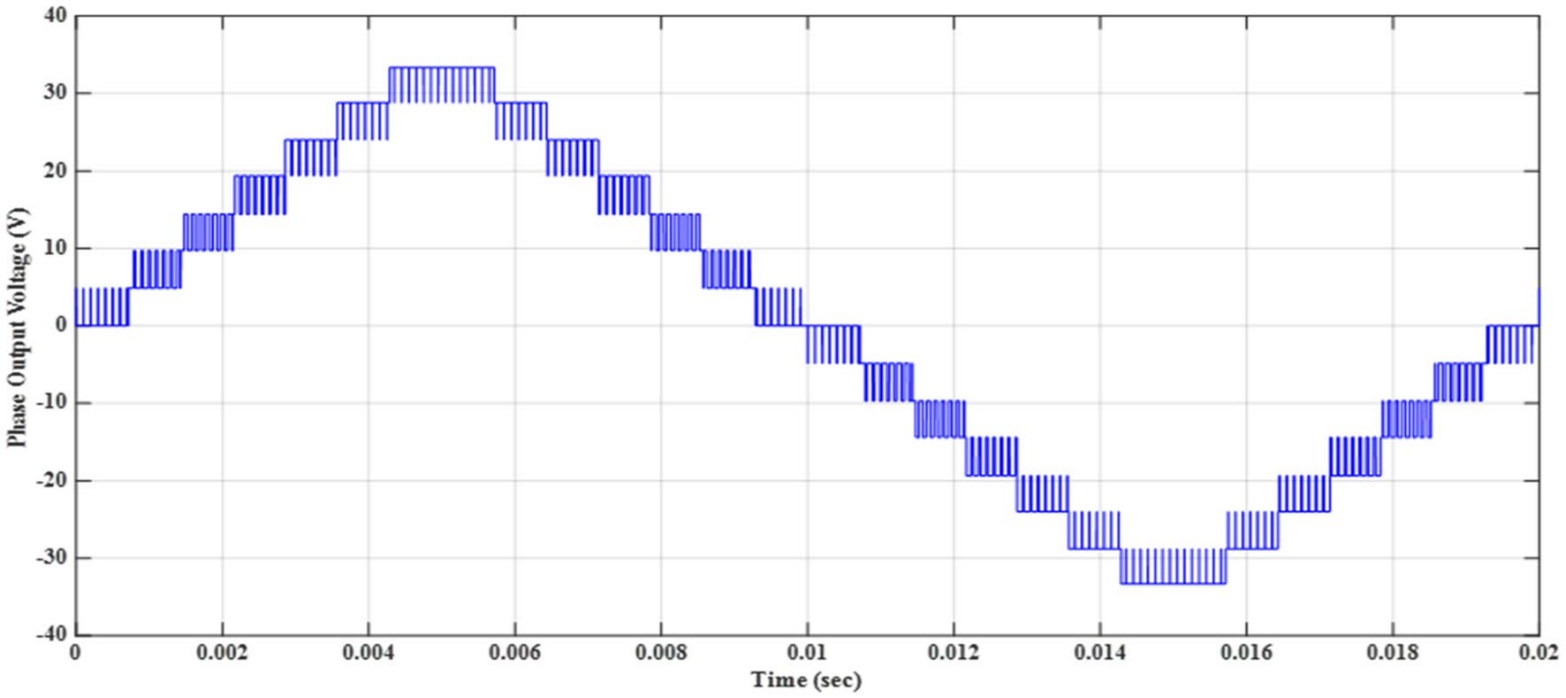
Output voltage for 15-level inverter using modified SPWM before implementing optimization.

In this method, the waveform is divided into $(m-1)$ sectors, where ‘$m$’ represents the number of output voltage levels. Each sector corresponds to a specific combination of switching states for the inverter switches that determine the resultant voltage. Figure 6 illustrates dividing the waveform into sectors for a 5-level inverter and Fig. 7 shows the fifteen-level output voltage waveform before implementing optimization techniques.

**Figure 8:**
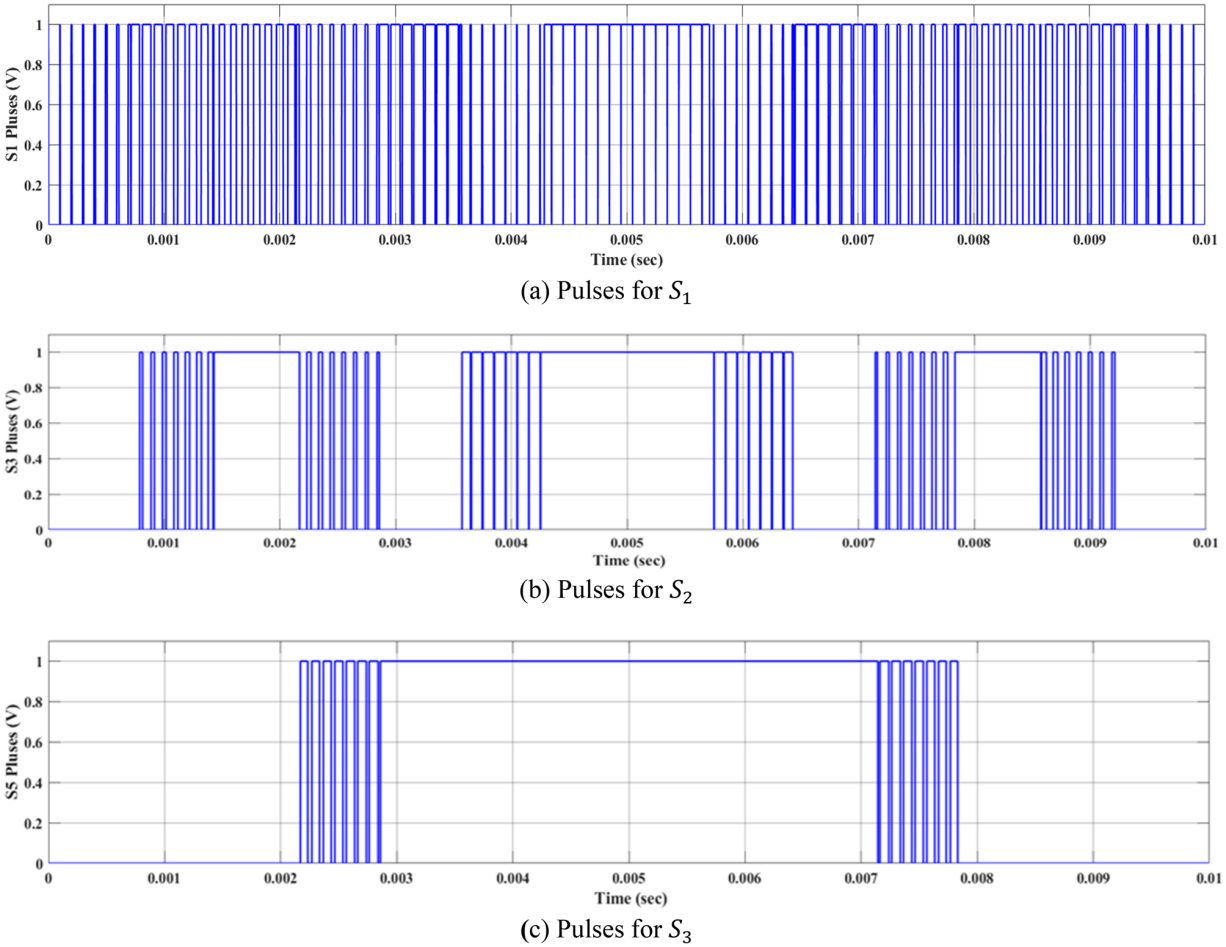
Resultant pulses for main switches at ${m}_{a}$ = 1.

Using the specified switches ${S}_{1}$, ${S}_{3}$, and ${S}_{5}$, which are the main switches in the fifteen-level inverter, the desired output-voltage levels can be achieved. The switches ${S}_{1}$, ${S}_{3}$, and ${S}_{5}$ are fired according to the resultant pulses generated by the technique as shown in Fig. 8. The firing patterns and combinations of switches may vary based on the sector and the desired output voltage level. The modified SPWM technique simplifies the pulse generation process. It improves the control of the MLI switches, resulting in high-quality output-voltage waveforms with reduced harmonic content and reduced complexity in the control of MLI switches.

**Figure 9:**
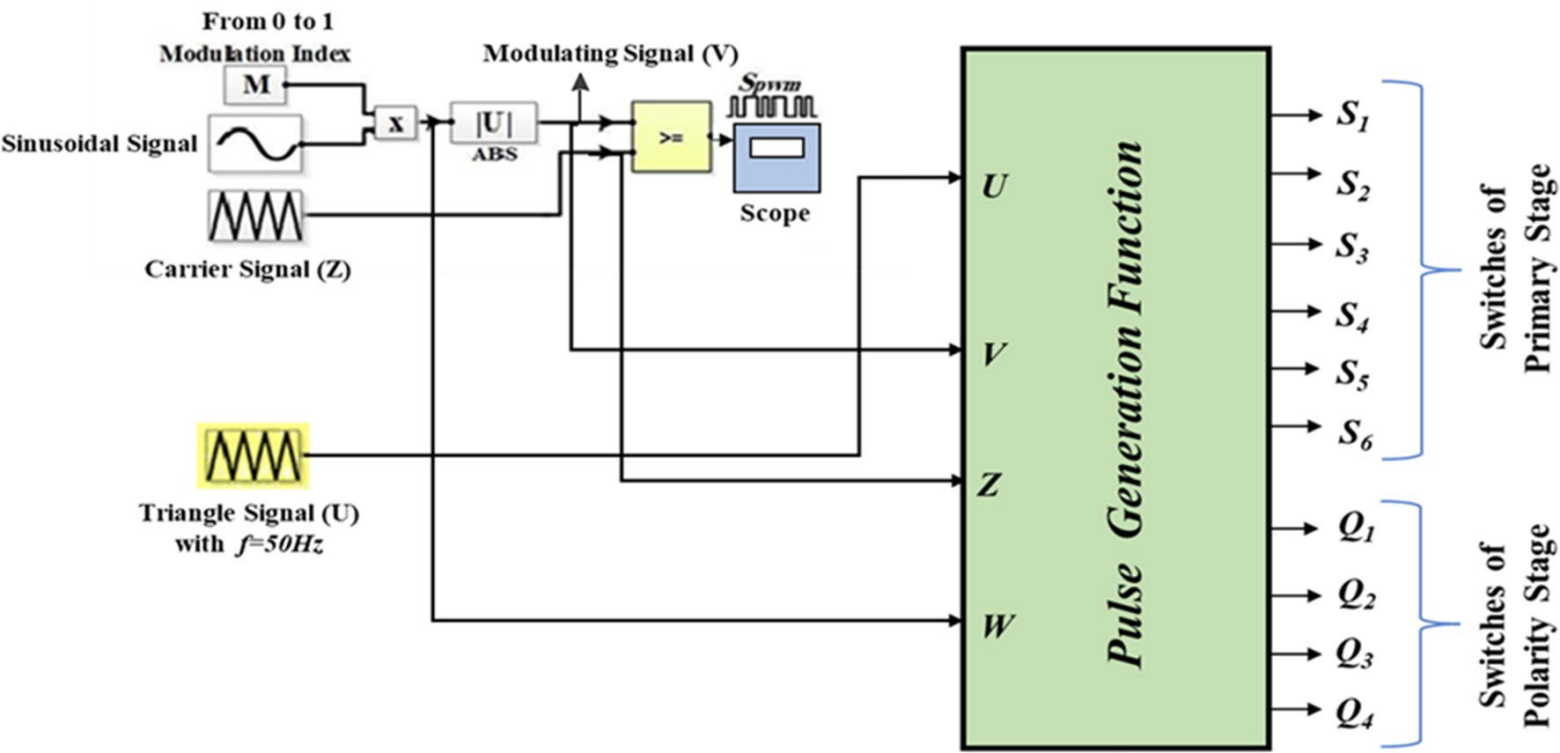
Simulation patterns designed for 15-Level MLI.

**Figure 10:**
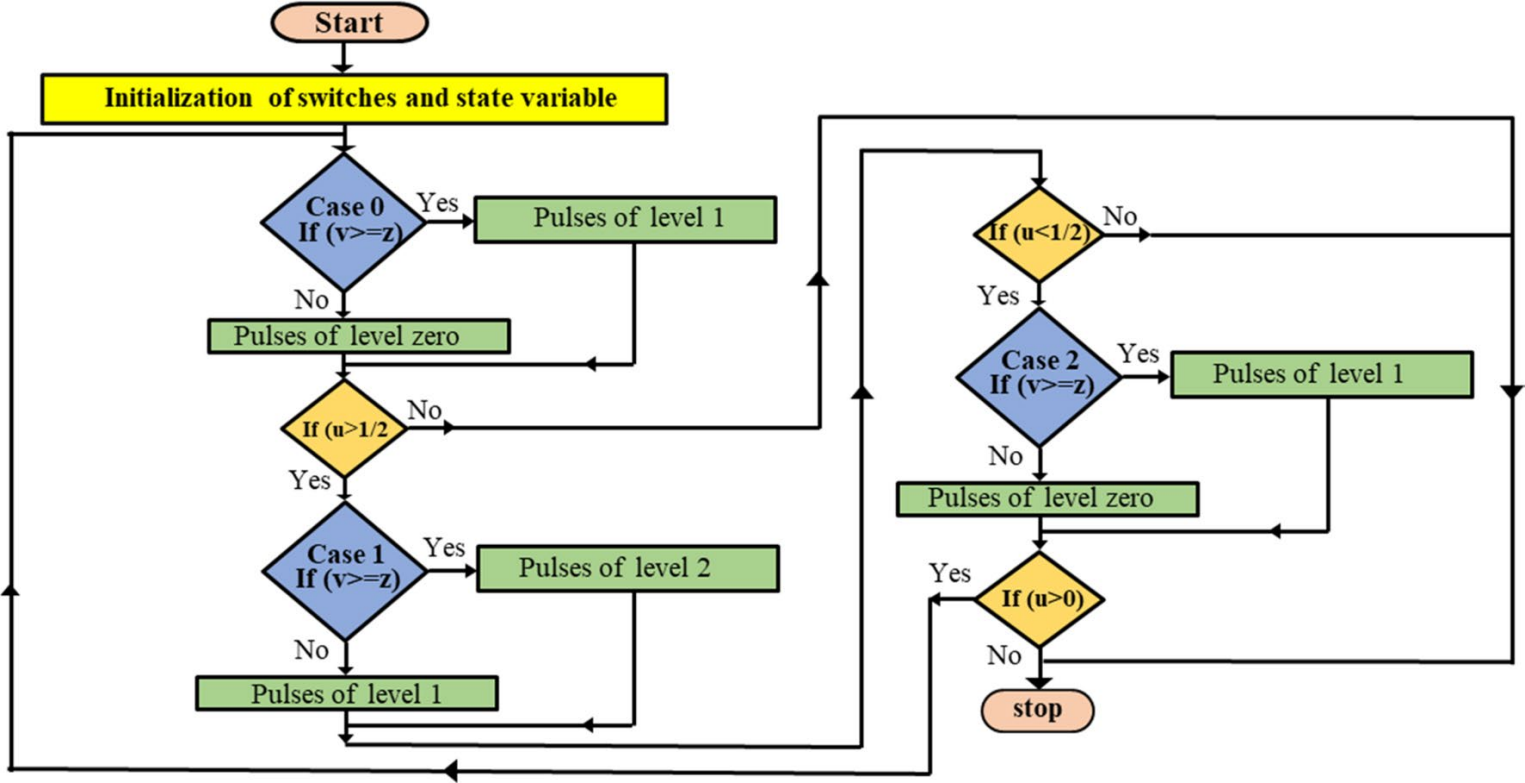
Flowchart of the proposed modified SPWM.

In this method, a designed MATLAB code is implemented to enable the connection between the pulse- generation process and the switches. The designed MATLAB-function block is shown in Fig. 9 while the MATLAB code is designed according to the flowchart indicated in Fig. 10.

Figure 9 shows a MATLAB function block. This block is designed by implementing the MATLAB/Simulink software to encapsulate a specific algorithm or functionality. This function block implements the pulse generation process based on the desired modulation technique, such as SPWM. The code implemented in the MATLAB function block considers the required inputs, such as the modulating signal and the carrier signal. It performs the necessary calculations and comparisons to generate the switching pulses. The designed MATLAB code can be found in Online Appendix B. These pulses are then connected to the switches within the inverter system. Figure 10 presumably represents a flowchart that outlines the steps involved in the pulse generation process. It represents the algorithm or logic implemented within the MATLAB function block to generate the switching pulses based on the modulation technique. A systematic approach was employed to investigate the effect of varying the modulation index on the values of the THD. The value of ${m}_{a}$ was entered through the block labeled ‘m’ as shown in Fig. 9. The model was then executed to obtain the corresponding THD value, which was reported. This process was repeated multiple times to cover the range of m, from 0 to 1, allowing for a comprehensive analysis of the relationship between modulation index and THD.

**Figure 11:**
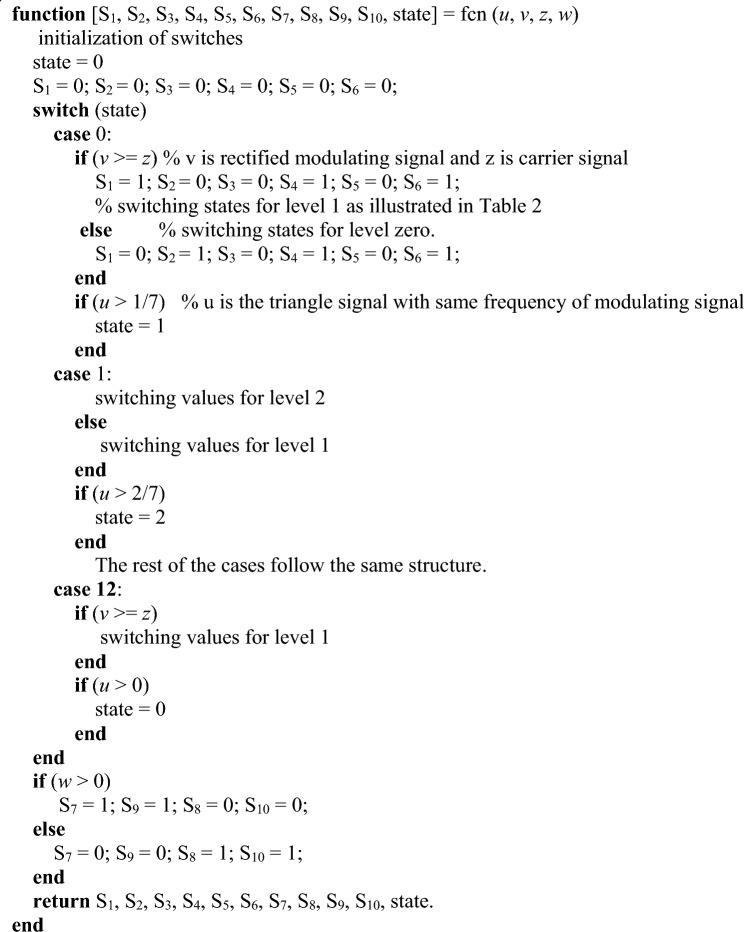
Pseudo-code of the modified SPWM technique.

The pseudocode provided in Fig. 11 represents a mathematical model for controlling the CHB-MLI. For CHB-MLI, the pseudocode outlines a control technique that governs the switching behavior of the CHB-MLI based on certain input parameters. The control technique employs a state-based approach, where the state variable “state” determines the current operating state of the inverter. The pseudocode defines a set of conditions and switches (*S*_1_–*S*_6_) for each state, which dictates the desired configuration of the H-bridge switches in the CHB-MLI. These switches control the generation of the desired AC output waveform. It also considers additional input parameters, such as “*u*”, “*v*”, “*z*”, and “*w*” which likely represent specific control signals or system variables. These parameters influence the decision-making process within each state and help determine the appropriate switching configuration for the inverter. Furthermore, the pseudocode includes an output section that assigns values to variables (*S*_*1*_ to *S*_*10*_ and *S*) based on the current state and input parameters. These variables can be used to monitor and analyze the inverter’s behavior or further control processes.

HSFM, known for its suitability in low-power applications and ability to shift low-order harmonics, proves advantageous by reducing THD through implementing a compact-sized filter. The SPWM technique is classified into the Phase-Shifted Modulation (PSM) and the Level-Shifted Modulation (LSM). The PSM employs multiple carrier signals with a phase shift, while LSM involves level-shifting the carriers. Other commonly employed control techniques in various MLI topologies encompass the SHE-PWM and the SV-PWM. However, when considering the specific configuration of the CHB-MLI discussed in this paper, traditional modulation techniques may not be directly applicable due to the disparity between the required number of carrier signals and available operating switches. The proposed modified SPWM technique simplifies the modulation scheme, necessitating only one carrier signal for any inverter level count. The significant reduction in the number of carriers not only facilitates practical implementation but also reduces overall system complexity. This method allows for easy integration with optimization codes, as it relies on code-based implementation. The proposed technique offers a practical solution for controlling CHB-MLIs, surmounting limitations, and streamlining complexity in MLI control systems. In the context of the CHB-MLI, traditional modulation techniques encounter a challenge due to the discrepancy between the required number of carrier signals and the available operating switches. Normally, the number of carrier signals needed equals the number of levels minus one. This difference in the number of carriers and operating switches poses a challenge when applying conventional modulation techniques. In contrast to traditional modulation techniques, the proposed SPWM technique overcomes the challenge mentioned above.

### GWO technique

GWO is a nature-inspired optimization technique that emulates the social hierarchy and hunting behavior of grey wolves. The idea behind GWO is to simulate the cooperative hunting behavior of grey wolves to solve complex optimization problems^44^. In GWO, a population of candidate solutions, represented as wolf packs, undergoes an iterative process to converge towards the optimal solution. The optimization process in GWO involves three main phases: encircling prey, hunting, and updating positions. Initially, the positions of the wolves, which represent potential solutions, are randomly initialized^45^. The encircling prey phase involves adjusting the positions of the wolves to surround the most promising solutions. This encircling behavior promotes the exploration of the search space. In the hunting phase, the wolves adjust their positions based on their own experience and the positions of other wolves. The alpha, beta, and delta wolves, which represent the best solutions found so far, guide the movement of the entire pack. This exploitation phase aims to converge toward the optimal solution by leveraging the information shared among the wolves^46^. The updating positions phase involves adjusting the positions of the wolves based on their prey’s fitness values. This allows the wolves to adapt and refine their positions to improve the overall performance of the pack.

The behavior of the GWO algorithm can be mathematically formulated to describe the hunting process of grey wolves. In this formulation, the alpha ($\alpha$) wolf position represents the best solution found so far, while the beta ($\beta$) and delta ($\delta$) positions represent the second and third best solutions, respectively. The rest of the solutions are represented by the omega ($\omega$) positions^12^.

During the hunting process, the $\alpha$, $\beta$, and $\delta$ wolves guide the movement of the $\omega$ wolves. The attacking process of the grey wolves involves several steps, starting with encircling the prey to prevent it from escaping. This encircling behavior can be represented by Eqs. (8) and (9).

8 $${D}^{\to }=\left|{C}^{\to }. {X}_{p}^{\to }(i)-{X}^{\to }(i)\right|$$

9 $${X}^{\to }(i+1)= {X}_{p}^{\to }(i)-{A}^{\to }.{D}^{\to }$$

where ${A}^{\to }$ and ${D}^{\to }$ are vector coefficients, $i$ is the current iteration, and ${X}^{\to }$ is a grey wolf’s location vector. Finding ${A}^{\to }$ and ${C}^{\to }$ vectors yield the encircling Eqs. (10) and (11).

10 $${A}^{\to }=2{a}^{\to }.{r}_{1}^{\to }-{a}^{\to }$$

11 $${C}^{\to }=2.{r}_{2}^{\to }$$

In Eq. (12) of GWO algorithm, the variable “$a$” represents the coefficient that decreases linearly from 2 to 0 over the course of the iterations. It is used to update the position of the search agents.

12 $$a=2-i*(\frac{2}{max\_iter})$$

Here, $a$ is a scalar value that controls the exploration–exploitation balance of the algorithm. It decreases linearly with the current iteration number “$i$”and the maximum number of iterations “$max\_iter$” and ${r}_{1}$ and ${r}_{2}$ are random values between (0, 1).

The best $\alpha$, $\beta$, and $\delta$ solutions are saved, and the other wolves ($\omega$) update their positions based on the best solutions. The equations from (13) to (15) represent these steps.

13 $${D}_{Alpha}^{\to }=|{C}_{1}^{\to }.{X}_{Alpha}^{\to }-{X}^{\to }|$$

14 $${D}_{Delta}^{\to }=|{C}_{3}^{\to }.{X}_{Delta}^{\to }-{X}^{\to }|$$

15 $${D}_{Beta}^{\to }=|{C}_{2}^{\to }.{X}_{Beta}^{\to }-{X}^{\to }|$$

The vector positions of the wolves can be calculated using the following Eqs. From (16) to (18) based on the $\alpha$, $\beta$, and $\delta$ positions.

16 $${X}_{1}^{\to }=|{X}_{Alpha}^{\to }-{A}_{1}^{\to }.{D}_{Alpha}^{\to }|$$

17 $${X}_{2}^{\to }=|{X}_{Beta}^{\to }-{A}_{2}^{\to }.{D}_{Beta}^{\to }|$$

18 $${X}_{3}^{\to }=\left|{X}_{Delta}^{\to }-{A}_{3}^{\to }.{D}_{Delta}^{\to }\right|$$

In these equations, ${C}_{1}^{\to }$, ${C}_{2}^{\to }$, and ${C}_{3}^{\to }$ are random coefficients generated within the range [0, 2]. ${A}_{1}^{\to }$, ${A}_{2}^{\to }$, and ${A}_{3}^{\to }$ are random coefficients generated within the range [-$a$, $a$], where $a$ is the linearly decreasing coefficient defined in Eq. (12).

Finally, Eq. (19) computes the updated position of the search agent by taking the average of ${X}_{1}^{\to }$, ${X}_{2}^{\to }$, and ${X}_{3}^{\to }$.

19 $${X}^{\to }(i+1)=\frac{({X}_{1}^{\to }+{X}_{2}^{\to }+{X}_{3}^{\to })}{3}$$

Equation (19) calculates the new position for each coordinate (dimension) of the search agent based on the updated values of ${X}_{1}$, ${X}_{2}$, and ${X}_{3}$. These equations are used to update the positions of the search agents iteratively, allowing GWO to explore and exploit the search space to find optimal solutions.

### Proposed control objective and optimal-tuning implementation

Optimization techniques play a vital role in power electronics applications, allowing for the efficient design and control of various systems. These techniques utilize mathematical algorithms and models to optimize parameters and achieve desired performance objectives. In power electronics, optimization is crucial for converter design, power management, control strategies, and energy efficiency improvement. Optimization methods like GA, PSO, GWO, and gradient-based approaches are commonly employed to tackle challenges such as maximizing power conversion efficiency, minimizing losses, optimizing switching frequencies, and achieving optimal sizing of components. These techniques help engineers and researchers in power electronics to find optimal solutions that balance conflicting objectives, enhance system performance, reduce costs, and meet stringent requirements. By leveraging optimization techniques, power electronics systems can be optimized to deliver improved power quality, higher reliability, and increased energy efficiency. These methods contribute to advancements in RE integration, electric vehicle technology, smart grids, and other power electronic applications^47^. The proposed objective, the GWO technique implementation, and the implementation of the proposed control scheme are discussed in the following sub-sections. Also, the GA and PSO optimization techniques are implemented for comparison.

### Proposed control objective

The main goal of different optimization techniques is to achieve the optimal width for each voltage level and minimize the THD indicated by Eq. (20).

20 $$THD=\frac{\sqrt{\sum_{n=\mathrm{3,5},7...}^{\infty }{{V}_{n}}^{2}}}{{V}_{1}}$$

where ${V}_{n}$ is rms harmonics voltages, ${V}_{1}$ is rms fundamental voltage, and $n$ is number of harmonics.

To integrate the simulation program of the MLI model with the optimization algorithms, a MATLAB environment is used. The objective function needs to be satisfied. The condition requires the sector values to be in ascending order and within the range of 0 and 1.

### Implementation of the proposed control scheme

**Figure 12:**
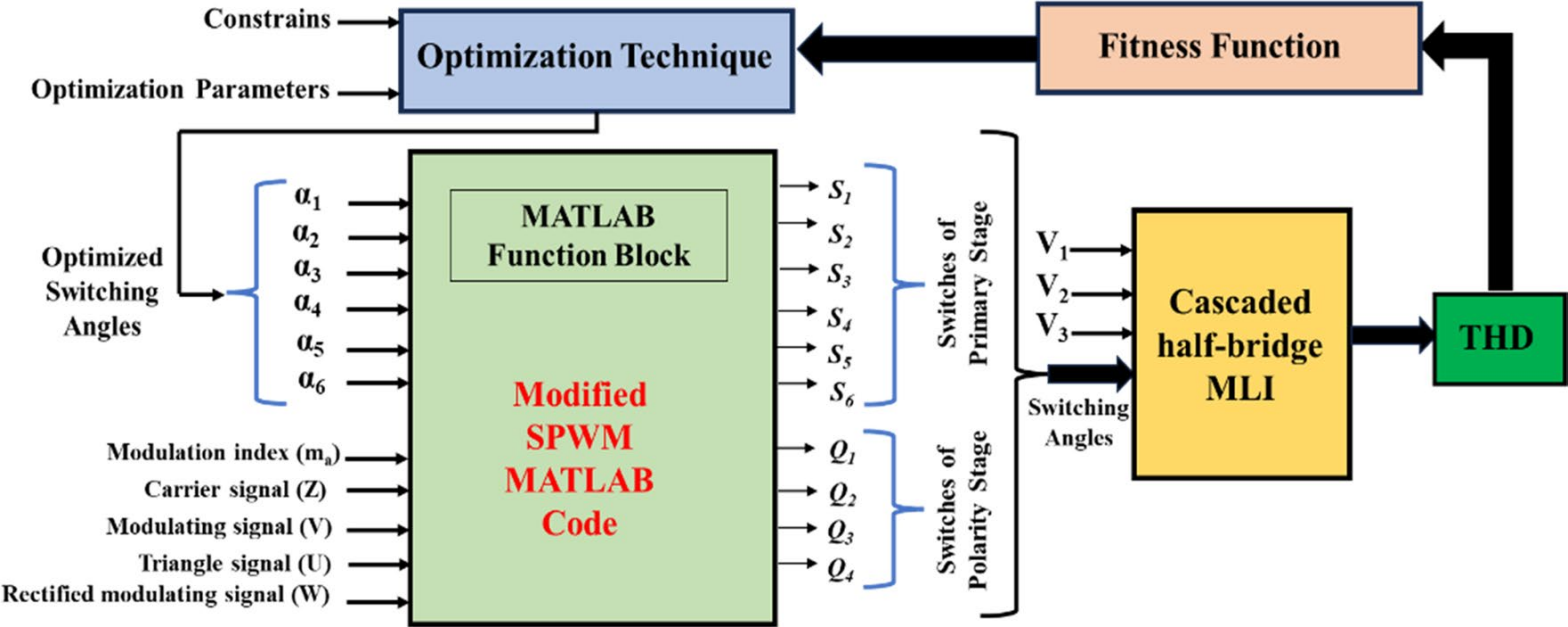
Complete model of switching for CHB-MLI.

Figure 12 demonstrates the complete model employed to determine the switching angles for the MLI. These angles are crucial for the MLI to generate the desired voltage waveform by appropriately switching the pulses and DC voltages. The pulse generation process is elucidated in Online Appendix B. It is implemented through a custom-designed code utilizing a MATLAB function block. The code requires three essential signals: the carrier signal, the modulating signal, and the triangle signal along with the modulation index value to execute the computation and generate the switching angles. The output-voltage waveform is then subjected to Fast Fourier Transform (FFT) analysis, enabling the calculation of THD using Eq. (20). Minimizing the THD value is imperative, necessitating the determination of optimal switching angles through optimization techniques like GWO, GA, and PSO. These techniques require the value of the output THD, and the specific parameters associated with each optimization algorithm. Furthermore, the constraints imposed on the optimization process are governed by Eqs. (21) and (22).

21 $$ \begin{gathered} 0 \le Sector_{1} \le 1 \hfill \\ 0 \le Sector_{2} \le 1 \hfill \\ 0 \le Sector_{3} \le 1 \hfill \\ 0 \le Sector_{4} \le 1 \hfill \\ 0 \le Sector_{5} \le 1 \hfill \\ 0 \le Sector_{6} \le 1 \hfill \\ \end{gathered} $$

22 $$Secto{r}_{1}<Secto{r}_{2}<Secto{r}_{3}<Secto{r}_{4}<Secto{r}_{5}<Secto{r}_{6}$$

The integration of these mathematical models, code implementation, and optimization techniques demonstrate a systematic approach to achieve minimal THD and optimal performance in the proposed control scheme. This comprehensive methodology is essential for scientific inquiry and practical implementation, ensuring accurate analysis and effective control strategies within the MLI system.

### Implementation of the GWO technique

**Figure 13:**
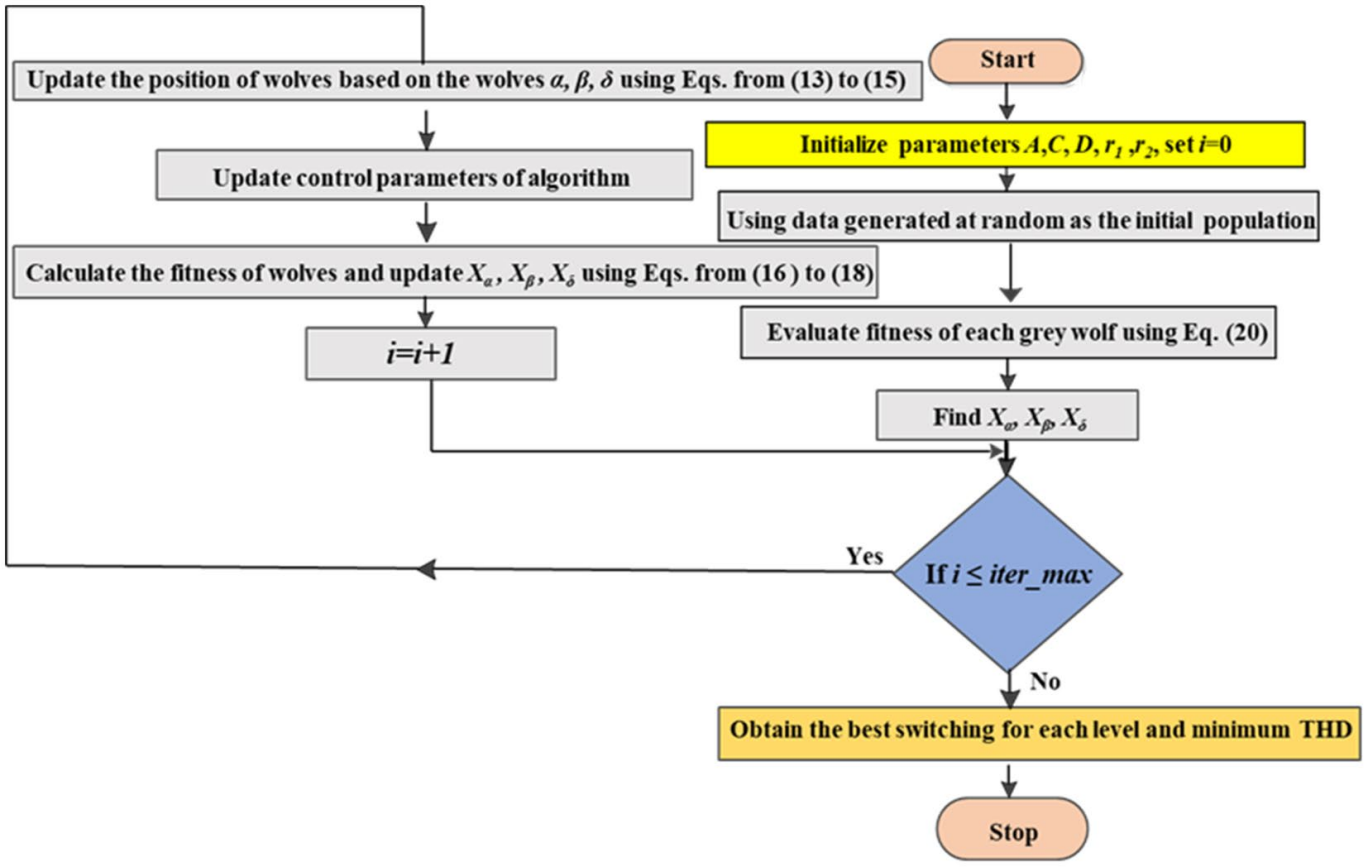
Flowchart of the GWO technique process.

The proposed modified-SPWM control scheme is implemented using MATLAB R2017b on a laptop equipped with an Intel Core i7-9750H CPU @ 2.60 GHz. Equation (20) described in the “Proposed control objective” sub-section denotes the fitness function to be optimized using the GWO technique to control the switching angles of the MLI. The GWO allows several advantages, including robustness, fast convergence, and minimal adjustment of its gains. The constraints of the firing angles for the MLI are firstly regulated using the modified PWM technique. These parameters are employed to produce the GWO Population. The initial population is generated using firing angle values and fitness is considered for each set of variables. The minimum-fitness variable is chosen as $\alpha$, $\beta$, and $\delta$. The lasting of the wolves is selected as $\omega$. The THD is the performance index to be minimized. The GWO algorithm presented in the “GWO technique” section with the designed MATLAB code included in Online Appendix C is employed to derive a single solution for each modulation index value. To encompass the entire range of modulation indices, the algorithm is executed iteratively across multiple runs. The GWO technique has been tracked for 10 self-governing trials implementing different settings until the required solutions are near enough to each other and the change of solution is negligible. The implemented GWO flow chart, which presents the main stages of the GWO method, is indicated in Fig. 13. According to several trials, the GWO’s factors implemented in the tuning process are shown in Table 3.

**Table 3:** parameters of the GWO algorithm.

Parameters	Values
Search agents	50
Number of dimensions	6
Random number (*r*_*1*_) range	[0, 1]
Random number (*r*_*2*_)* range*	[0, 1]
Maximum iterations	500

### Implementation of the GA technique

**Figure 14:**
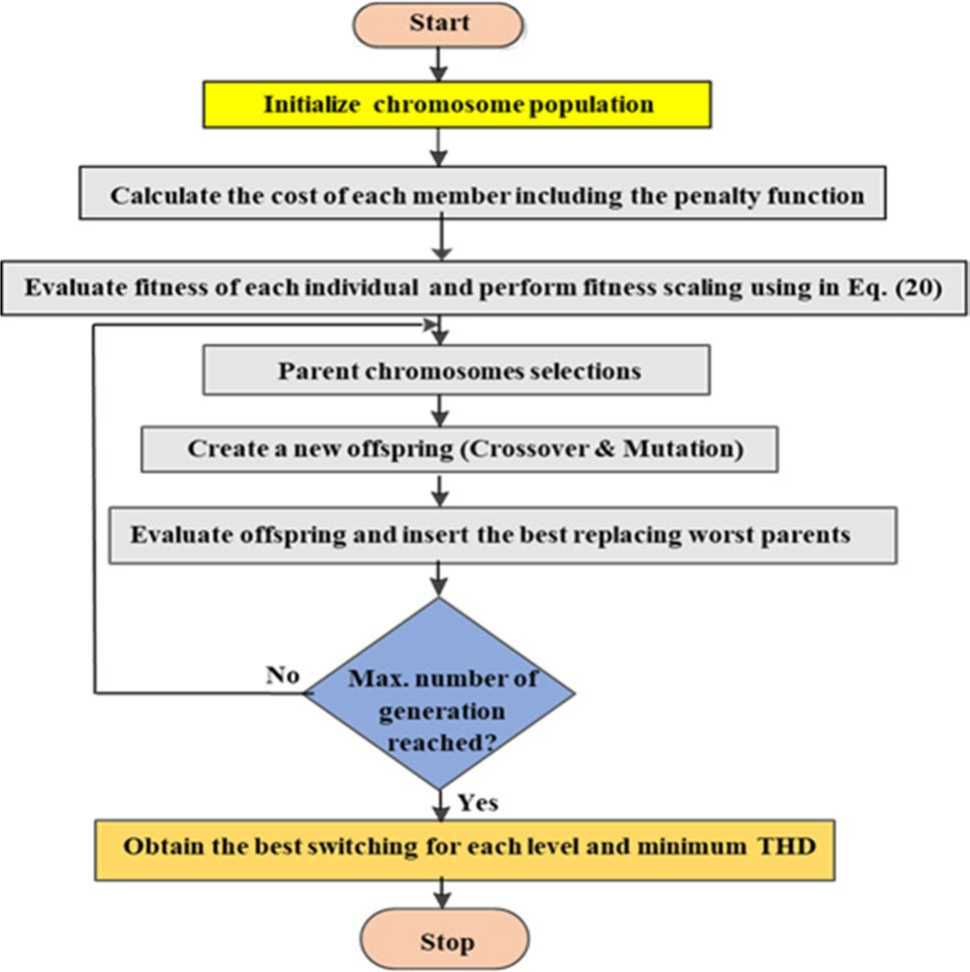
Flowchart of the implemented GA technique.

In this paper, The GA technique is used for comparison. The GA technique in^48^ is implemented to find the optimal switching angle of the proposed modified SPWM control scheme, described in the “Modified SPWM technique for MLI” section, implementing the fitness function indicated by Eq. (20). The starting population of chromosomes that implies the switching angles is randomly generated. Then, the objective function indicated by Eq. (20) in the “Proposed control objective” sub-section is used to calculate the chromosomes. Based on the objective function of individuals, a new population is selected. Crossover and mutation rate are used in memberships of the population to produce the new population^49,50^. The evaluation and generation process are automatically implemented until the predetermined termination condition has been satisfied^51,52^. According to experience and several trials, mutation should be accomplished with a small probability ranging from a value of 0.1–2%, while crossover should be selected to be in the range of 60–90%. The GA technique has been tracked for 10 self-governing trials implementing different settings until the required solutions are real near enough to each other. The implemented GA’s flow chart is presented in Fig. 14. According to different trials, the basic factors of the implemented GA are selected and given in Table 4.

**Table 4:** Parameters of the GA algorithm.

Parameters	Values
Population size	50
Number of generations	500
Number of dimensions	6
Number of elite individuals ($nElite$)	50
Cross over rate	0.8
Mutation rate	0.01

**Figure 15:**
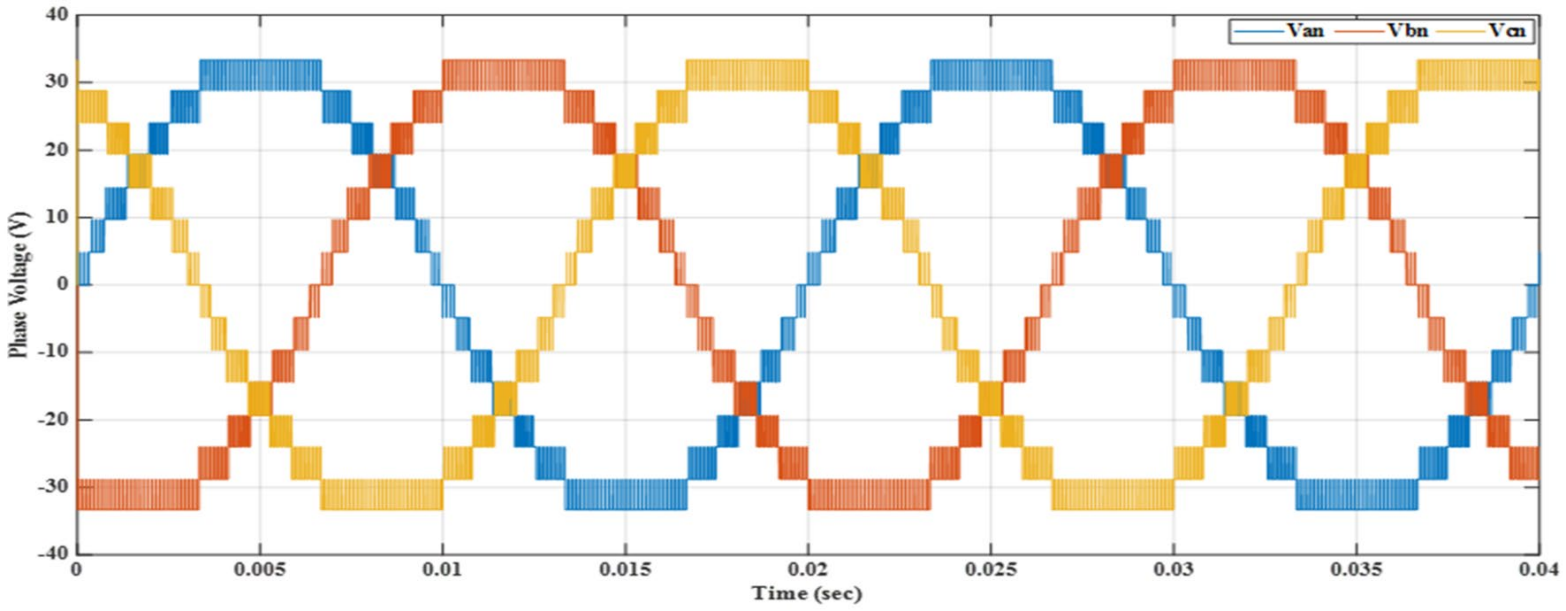
Phase voltages after implementing GA technique at ${m}_{a}=0.9$ and ${m}_{f}=200$.

Figure 15 shows the output voltage waveform for a three-phase system after applying the GA technique at a modulation index of 0.9. The waveform represents a balanced output voltage. The THD for this waveform was measured at 8.05727%.

### Implementation of the PSO technique

PSO operates by allowing particles to explore a multi-dimensional solution space in pursuit of an optimal solution. Each particle represents a potential solution to the optimization problem under consideration^53,54^. The movement of particles is governed by their velocities, which determine the direction and speed of their traversal through the solution space. By adjusting their velocities based on personal experience and the experiences of neighboring particles, the particles collectively navigate the solution space to seek improved solutions^55,56^.

**Figure 16:**
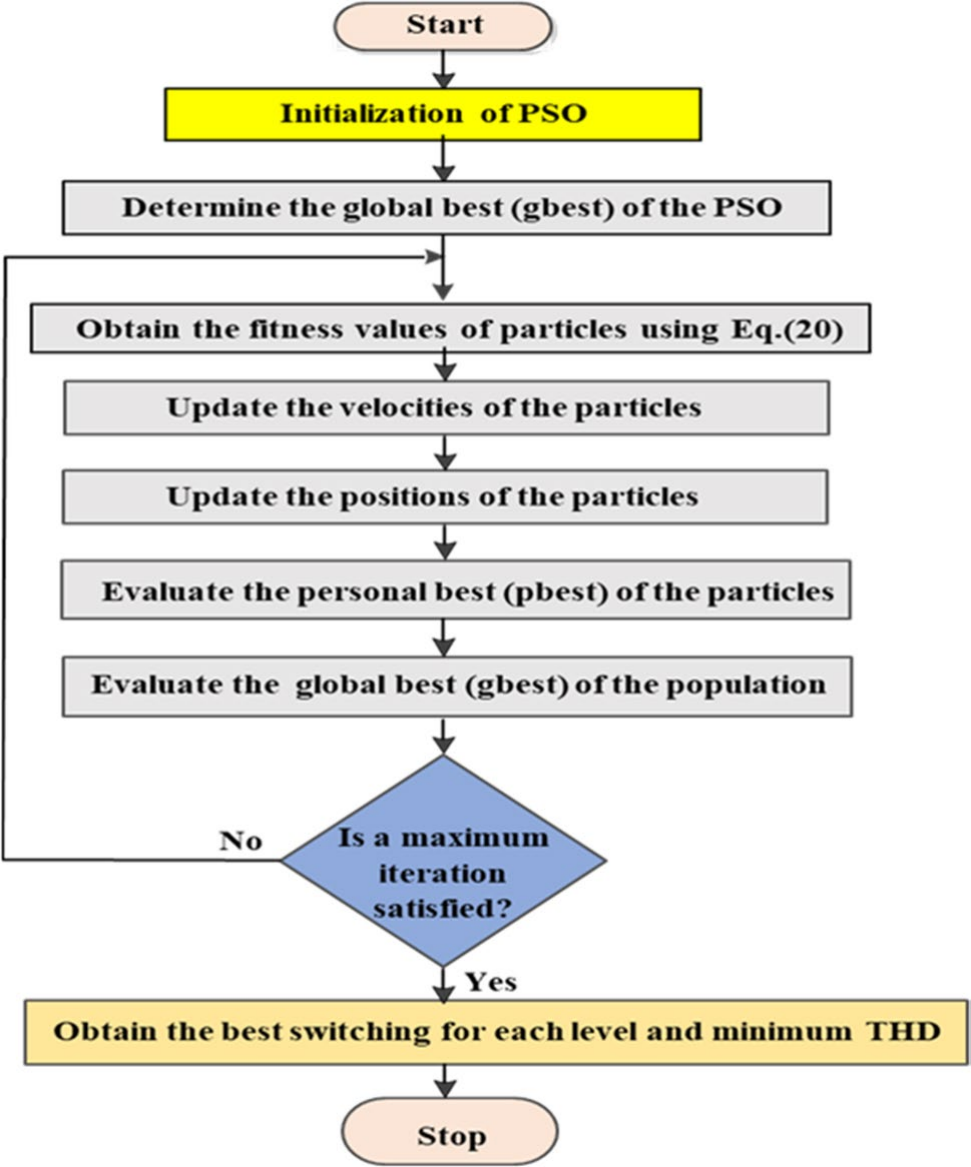
Flowchart of the PSO technique procedure.

In this paper, The PSO technique is used for comparison. The PSO discussed in^57^ is implemented to find the optimal switching angle of the proposed modified SPWM control scheme, described in the “Modified SPWM technique for MLI” section, implementing the fitness function indicated by Eq. (20). The PSO technique has been tracked for 10 self-governing trials implementing different settings until the required solutions are real near enough to each other. The implemented PSO’s flowchart is presented in Fig. 16. According to different trials, the basic factors of the implemented PSO are selected and given in Table 5.

**Table 5:** Implemented parameters of the PSO.

Parameters	Values
Population size	50
Number of dimensions	6
Number of generations	500
Acceleration constant, *C*_*1*_	1.5
Acceleration constant, *C*_*2*_	1.5
Initial inertia weight (*w*_*min*_)	0.2
Final inertia weight (*w*_*max*_)	0.9
Maximum velocity	6

**Figure 17:**
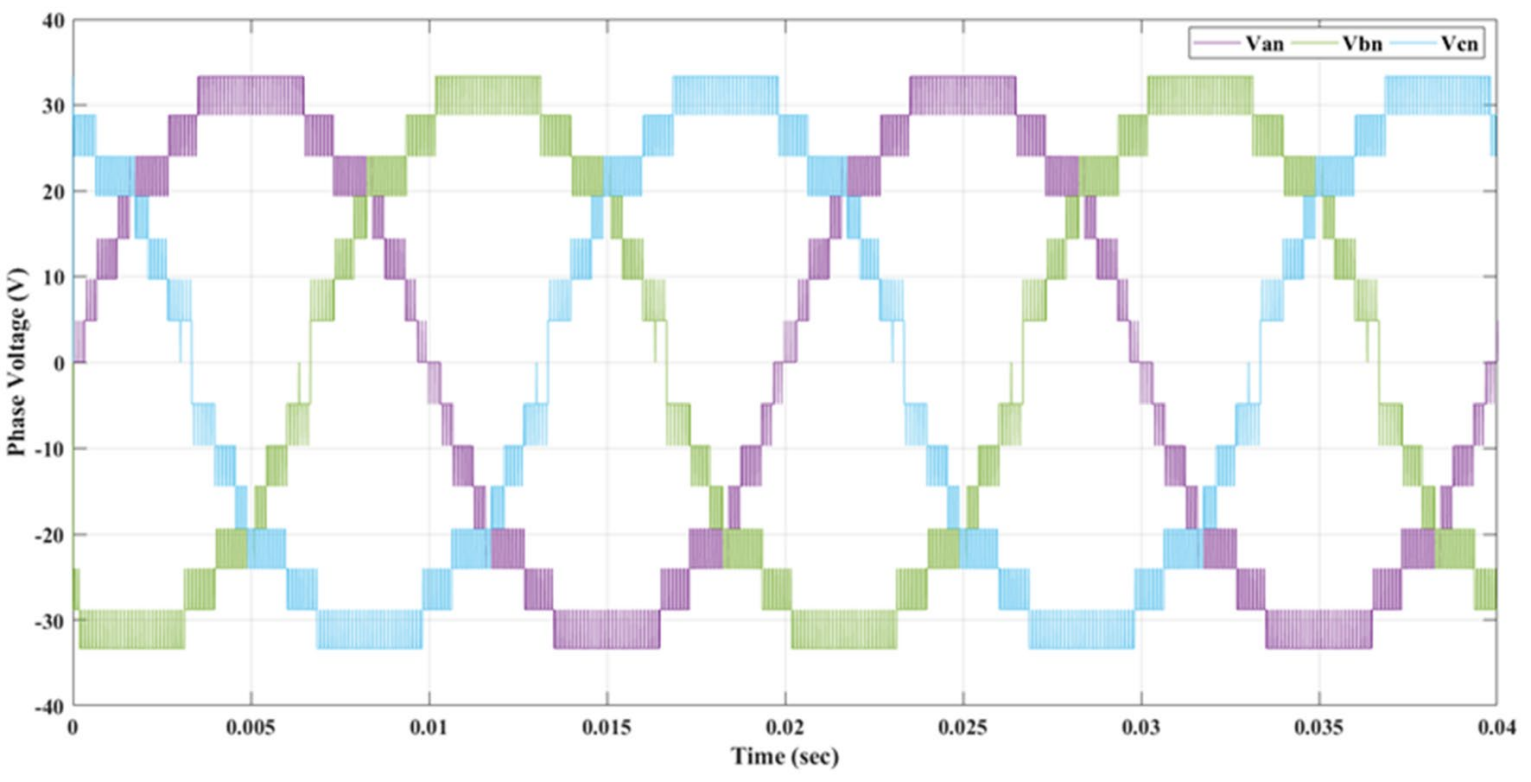
Phase voltages after implementing the PSO technique at ${m}_{a}=0.9$ and ${m}_{f}=200$.

The output-voltage waveform for the three-phase system, obtained by implementing the PSO technique at a modulation index of 0.9, is depicted in Fig. 17. The waveform exhibited in the figure demonstrates a balanced output voltage. The THD value for this waveform was quantified at 8.50726%.

## Simulation results

**Figure 18:**
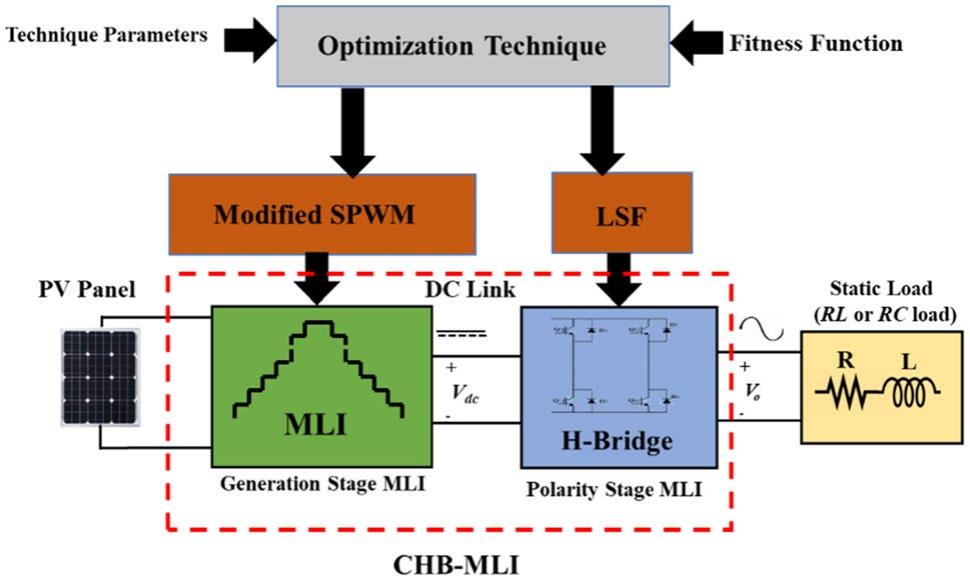
Complete system simulation model.

The complete system-simulation model depicted in Fig. 18 was implemented in MATLAB R2017b on a laptop equipped with an Intel Core i7-9750H CPU @ 2.60 GHz. The simulation was executed for a total runtime of one second. In the following subsections, the simulation results obtained using the modified SPWM technique will be investigated and analyzed. The optimal firing of switches, implementing the modified SPWM technique, is provided. The simulation results implementing the proposed modified SPWM control scheme for a three-phase half-bridge cascaded MLI is investigated. Additionally, the performance of the proposed control scheme is compared with the GA and PSO technique to determine its relative effectiveness.

### Results using modified SPWM

**Figure 19:**
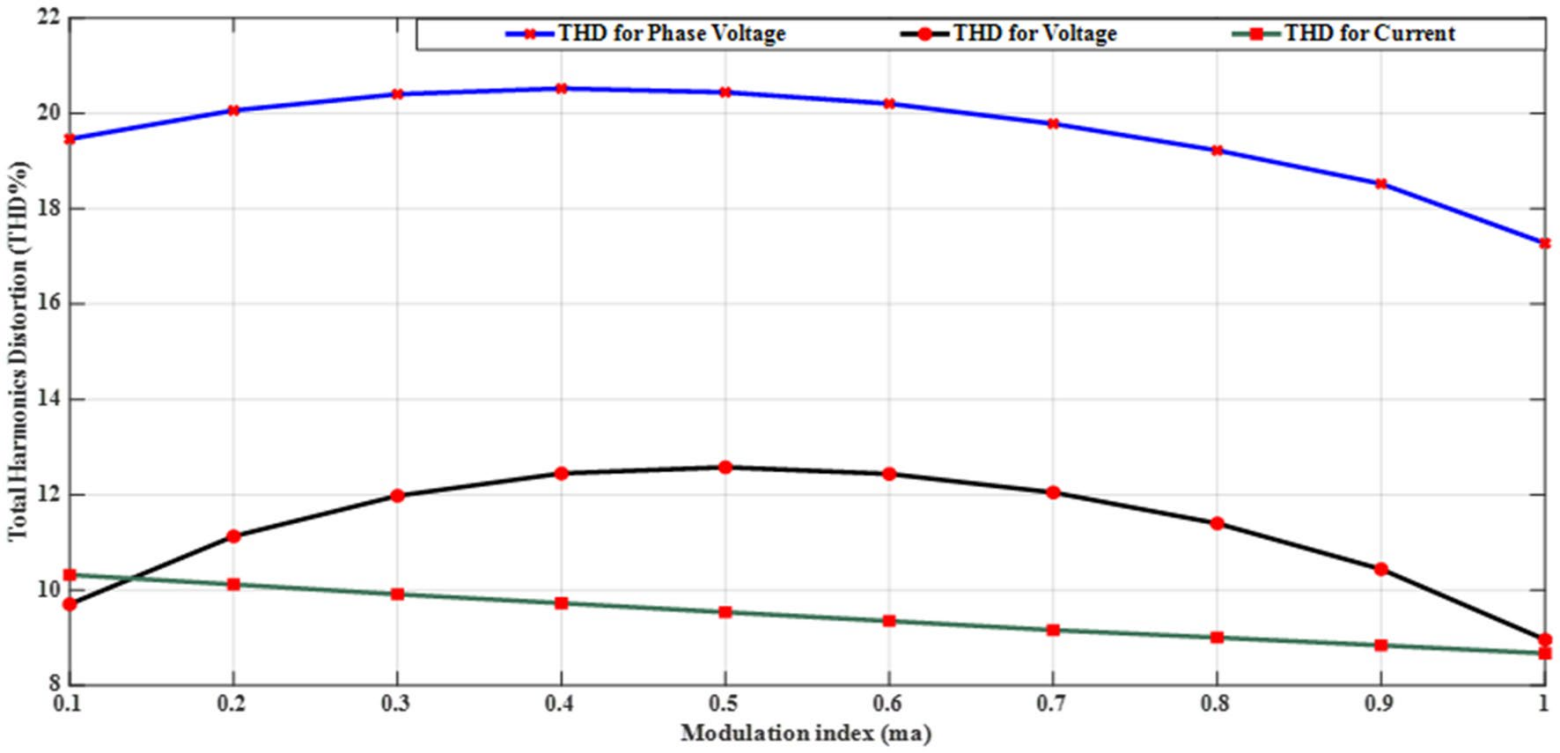
THD% versus ${m}_{a}$ for the modified SPWM technique.

Implementing the MATLAB/SIMULINK® simulation program, tests on the inverter are conducted by varying the modulation index (${m}_{a}$). The inverter is supplied with voltage of values ${V}_{1}=5\mathrm{ V}$, ${V}_{2}=10\mathrm{ V}$, and ${V}_{3}=20\mathrm{ V}$ operating at a frequency of ${f}_{m}$= 50 Hz. The inverter is designed to energize a resistive-inductive star connection system with a resistance per phase of ${R}_{load}=10\Omega$, an inductance per phase of ${L}_{load}=15\mathrm{ mH}$, and a carrier frequency of ${f}_{c}$= 10 kHz. Each level has the same width (equally stepped). Table 6 indicates the efforts of the THD of the phase and line voltages for different modulation indices. Figure 19 illustrates the relationship between the percent THD and the modulation index for the modified SPWM technique. One of the main advantages is the elimination of triplen harmonic components in the line voltage, resulting in a lower THD compared to the phase voltage.

**Table 6:** Variation of THD% versus ${m}_{a}$.

${m}_{a}$	0.1	0.2	0.3	0.4	0.5	0.6	0.7	0.8	0.9	1
$TH{D}_{v, ph}$	19.46	20.06	20.4	20.52	20.44	20.2	19.78	19.22	18.52	17.27
$TH{D}_{v, line}$	9.705	11.13	11.98	12.45	12.58	12.44	12.05	11.4	10.44	8.959
$TH{D}_{i}$	10.32	10.12	9.912	9.726	9.537	9.351	9.161	9.005	8.842	8.674

**Figure 20:**
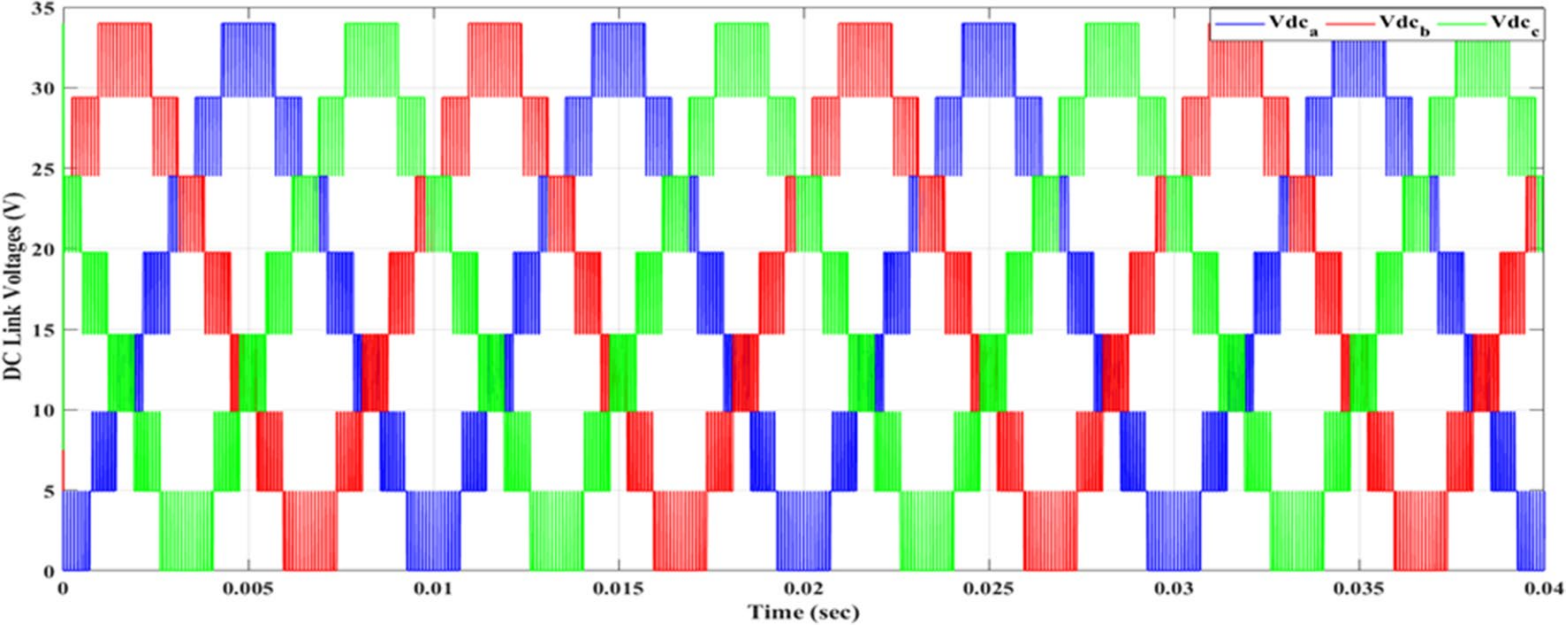
DC-Link voltages.

**Figure 21:**
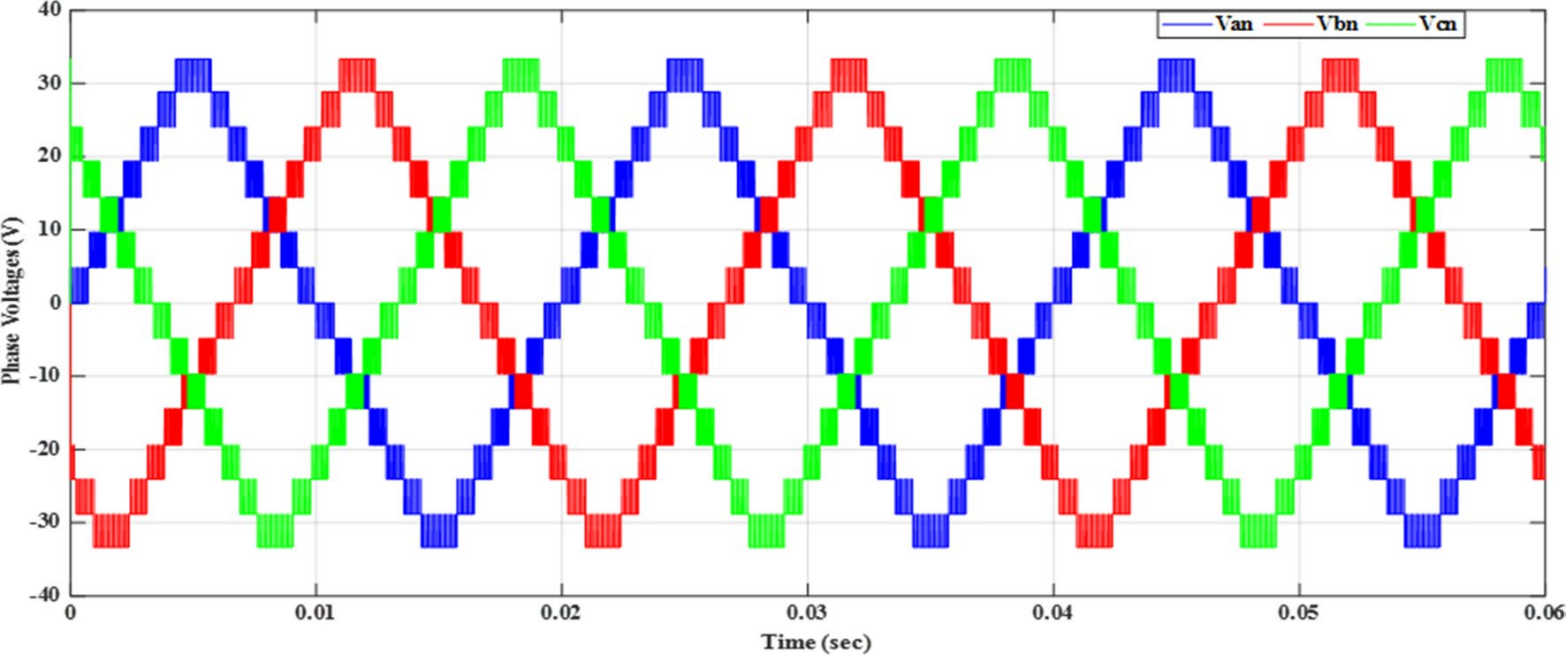
Three phase voltages with equally step before implementing optimization.

**Figure 22:**
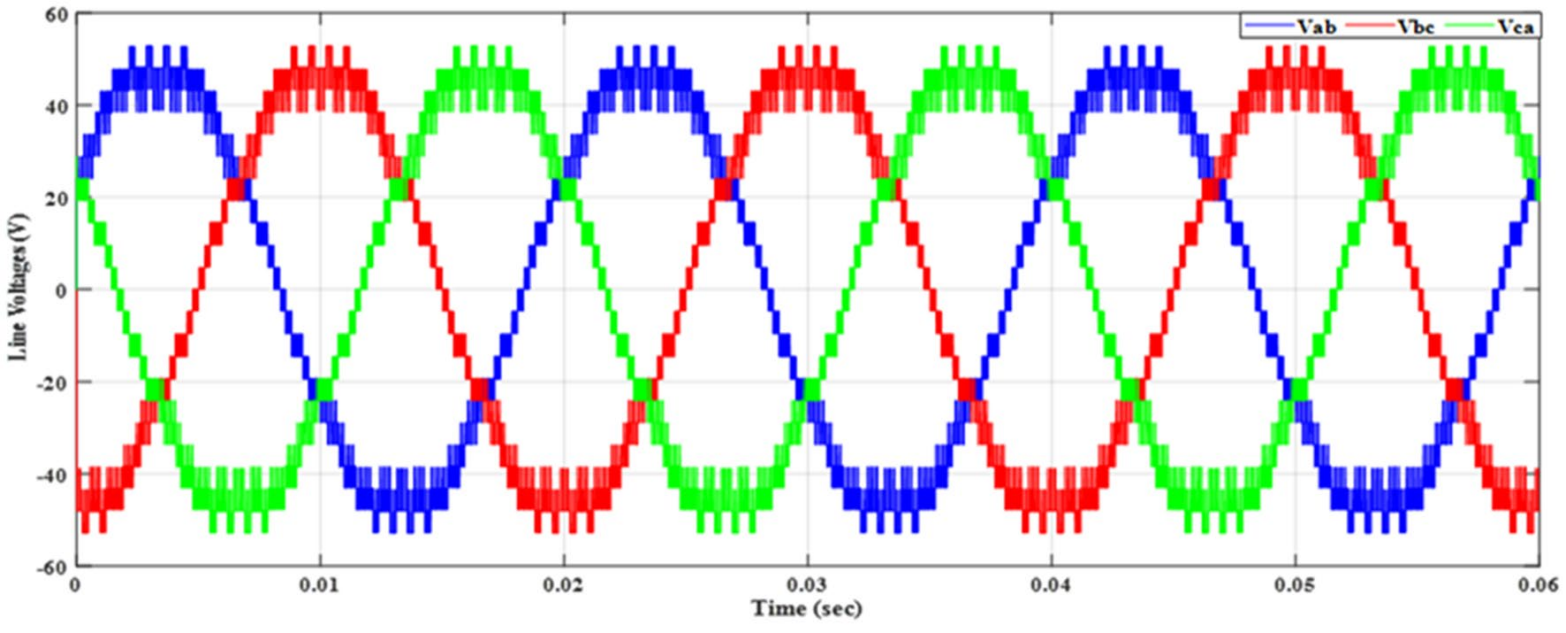
Line voltages before implementing optimization.

**Figure. 23:**
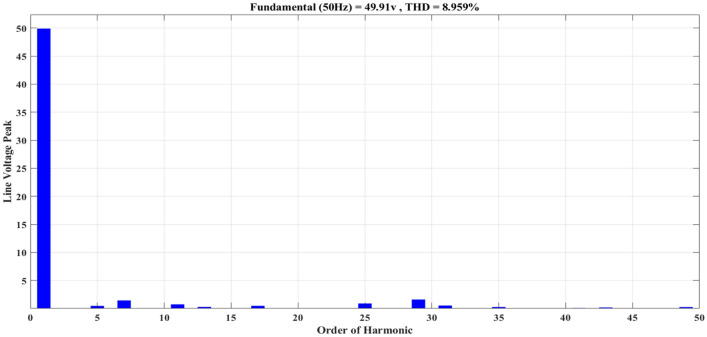
Harmonics spectrum of output line-voltage before implementing optimization.

**Figure. 24:**
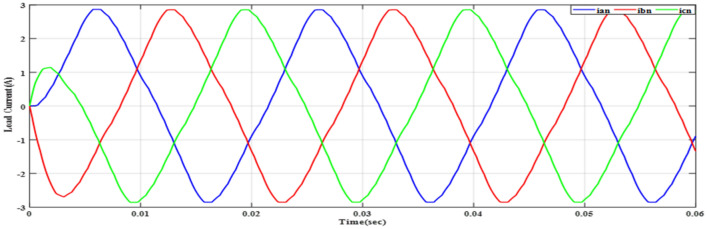
Line currents of RL load before implementing optimization.

Figure 20 depicts the stepped DC voltage produced by the inverter’s first stage, which is then converted into an AC voltage by the second stage. Both phase-output voltages and line voltages for the 15-level inverter are shown in Figs. 21 and 22 respectively. Figure 23 presents the corresponding FFT analysis for the line voltage of the 15-level inverter. It is obvious from Table 6, that the THD is 8.959% when ${m}_{a}$ is one. Figure 24 depicts the load currents for the case study.

The proposed scheme has undergone rigorous testing, involving the variation of the carrier signal frequency within a range of 5 kHz to higher values. Through meticulous analysis, it has been observed that this frequency modulation does not have a significant impact on the THD value. This finding underscores the robustness and stability of the scheme, indicating its ability to maintain consistent performance across different carrier signal frequencies. This outcome further strengthens the viability and reliability of the proposed scheme for its intended application.

### Results implementing GWO

**Figure 25:**
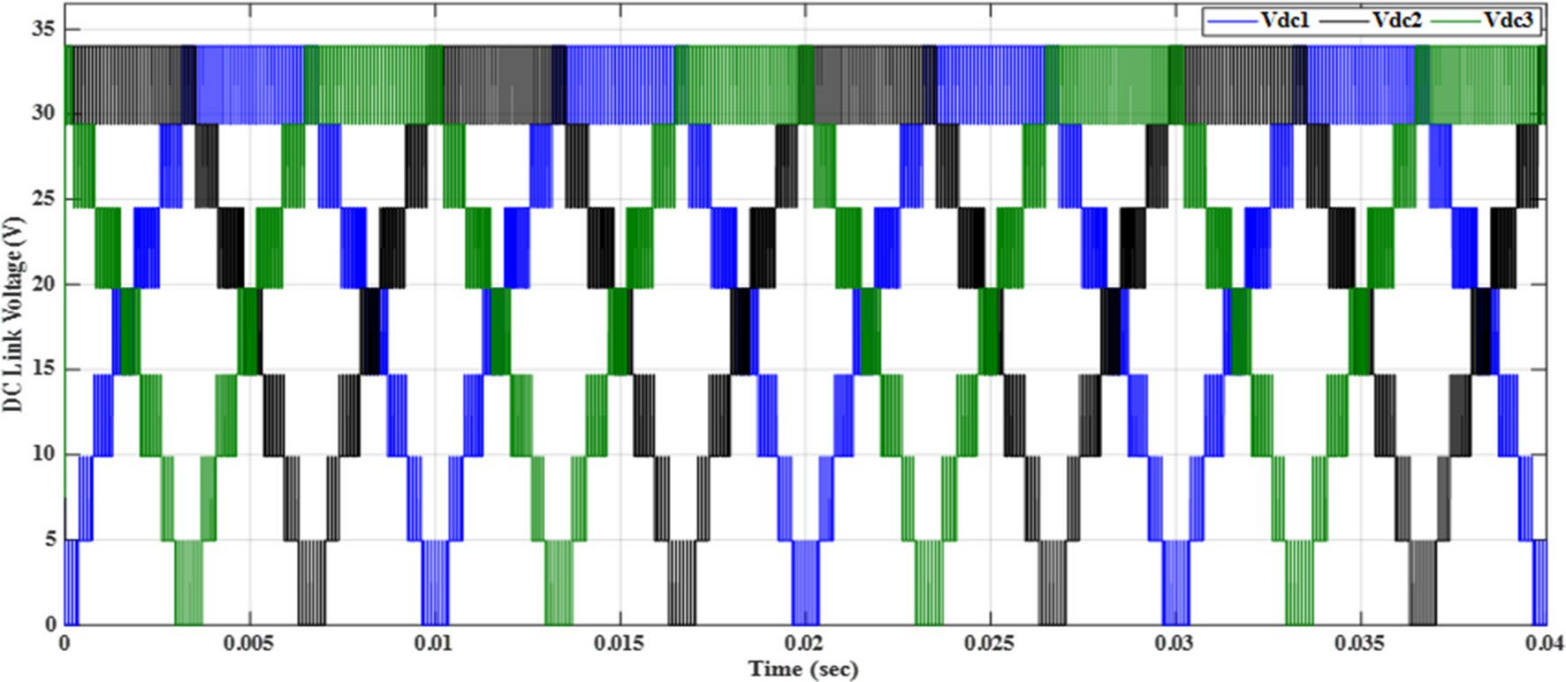
DC-Link voltages after optimization.

**Figure 26:**
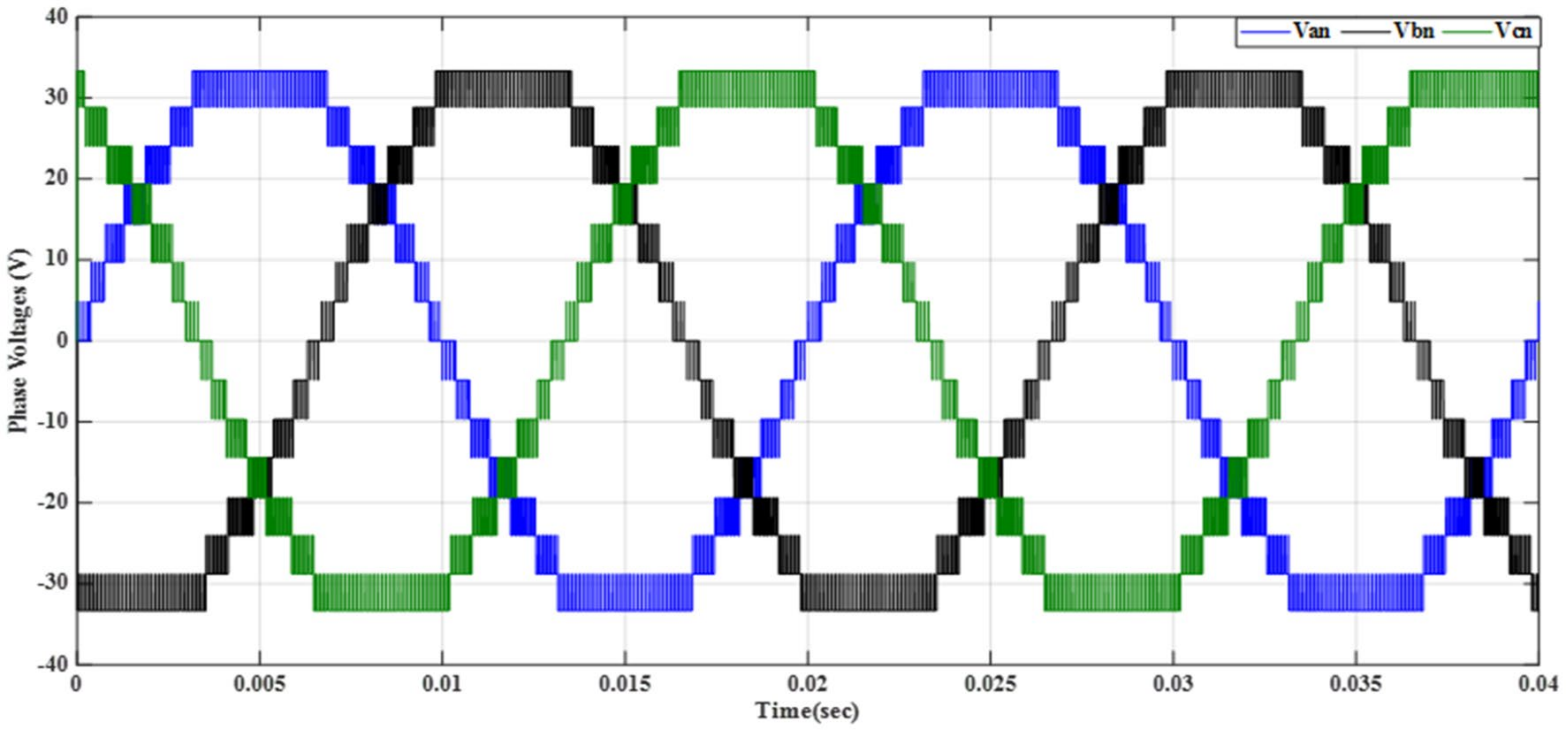
Phase voltages after implementing GWO technique at ${m}_{a}=0.9$ and ${m}_{f}=200$.

**Figure 27:**
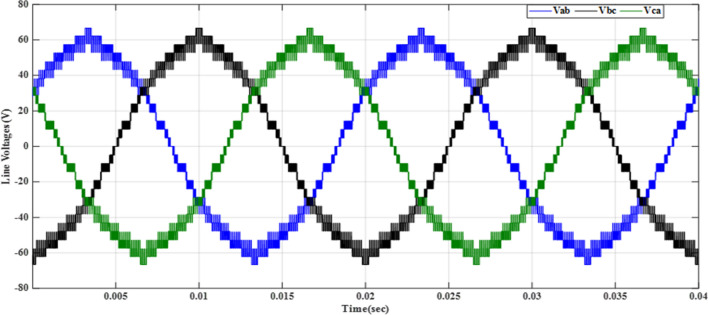
Line voltages after implementing GWO technique at same ${m}_{a}$ and ${m}_{f}$.

The MATLAB Simulink model is integrated with the designed code of the GWO algorithm presented in the “GWO technique” section and its implementation given in the “Implementation of the GWO technique” subsection. The simulation results presented in Table 7 are obtained by implementing the GWO algorithm with the specific parameters outlined in the accompanying Table 3. The relationship between the switching pattern and the modulation index for the fifteen levels is documented in Table 7. Furthermore, the observed outcomes include the waveforms of the output voltage from the first stage, three-phase voltages, and line voltages which are presented in the corresponding Figs. 25, 26, and 27, respectively. These waveforms represent a balanced three-phase system and exhibit a symmetrical distribution with equal magnitude between the three phases. This balance is essential for ensuring efficient power transmission and utilization within the system. The proposed scheme has undergone comprehensive testing to evaluate its performance under varying load conditions. To assess its ability to maintain optimal functionality, the load configuration was modified from an RL load to an RC load, with a resistance value ${R}_{load}=10\Omega$ and a capacitance value ${C}_{load}= 680 \mathrm{\mu F}$, resulting in a power factor (pf) of 0.9 leading. The outcomes of these tests, showcasing the scheme’s response to the changing load, have been presented in Table 7. These results provide valuable insights into the scheme’s adaptability and effectiveness in delivering consistent performance across different load configurations.

**Table 7:** Switching pattern for the fifteen levels using GWO Technique.

${m}_{a}$	Sector_1_	Sector_2_	Sector_3_	Sector_4_	Sector_5_	Sector_6_	$TH{D}_{v, ph}$	$TH{D}_{v, line}$	$TH{D}_{i,L}$	$TH{D}_{i,C}$
0.1	0.04827	0.14313	0.27297	0.37072	0.51721	0.67958	8.13747	7.48505	0.9888	8.922
0.2	0.02843	0.14895	0.24599	0.38337	0.52231	0.69577	9.38548	8.02109	1.1265	10.304
0.3	0.07453	0.14770	0.23988	0.34416	0.50525	0.66701	10.2237	8.5257	1.3191	11.194
0.4	0.05733	0.14081	0.23280	0.37535	0.52540	0.66417	10.5834	9.39443	1.4578	11.612
0.5	0.05813	0.14356	0.28291	0.35118	0.48279	0.64597	10.7251	9.78066	1.7362	11.774
0.6	0.0508	0.15024	0.28134	0.38887	0.53276	0.64414	10.8515	9.81647	1.2357	11.937
0.7	0.05080	0.15581	0.27726	0.36078	0.52262	0.64616	10.6553	9.64516	1.2255	11.698
0.8	0.05540	0.15443	0.26357	0.36587	0.51653	0.68797	10.2011	8.96454	1.4467	11.134
0.9	0.07115	0.14794	0.25821	0.36855	0.50735	0.62881	9.80255	8.42908	1.5323	10.732
1	0.07626	0.14667	0.26431	0.35873	0.49071	0.67475	9.04738	**6.87201**	0.9865	10.039

**Figure 28:**
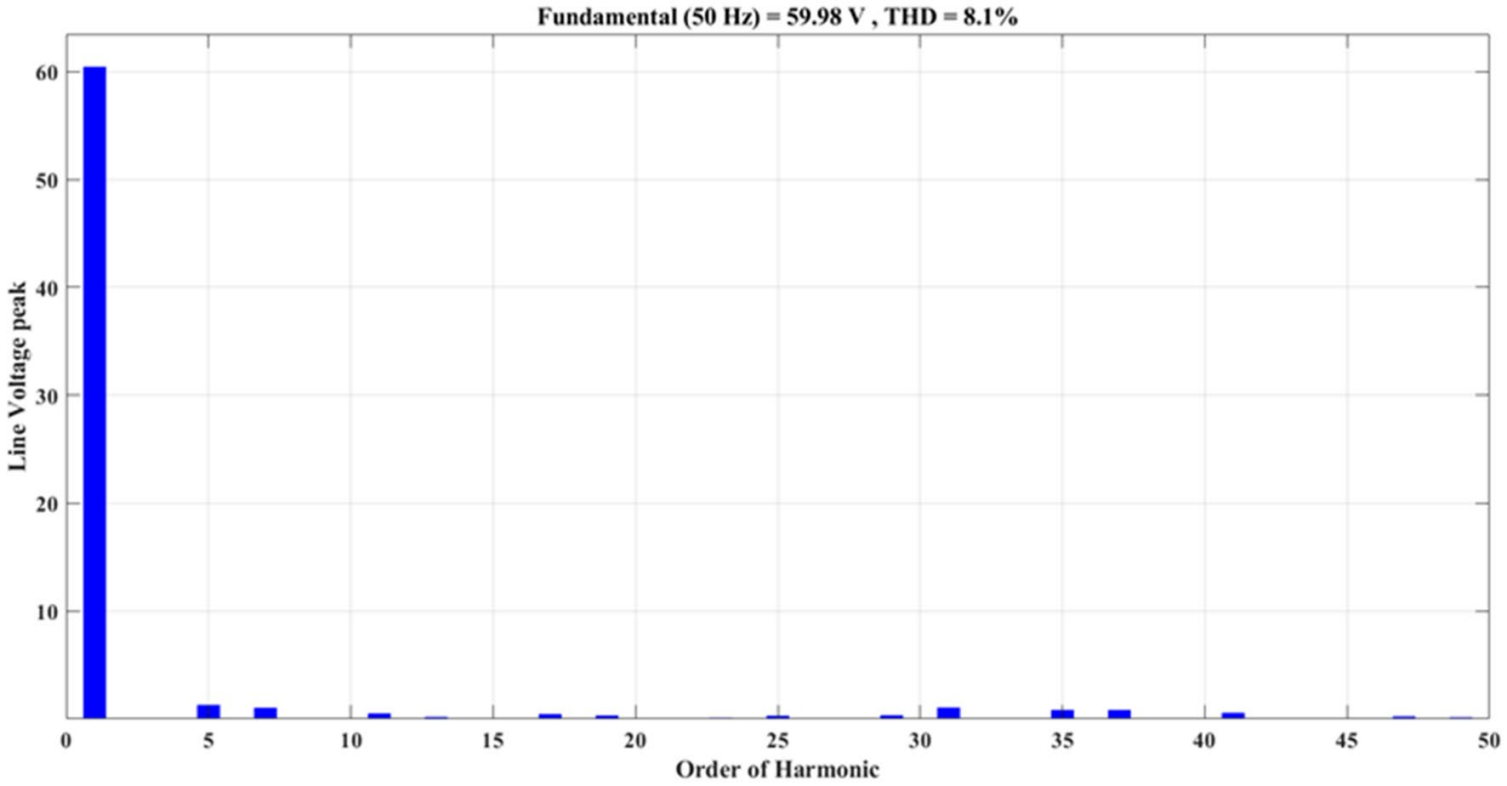
Harmonics spectrum of output line voltage implementing GWO at ${m}_{a}=0.9$ and ${m}_{f}=200$.

**Figure 29:**
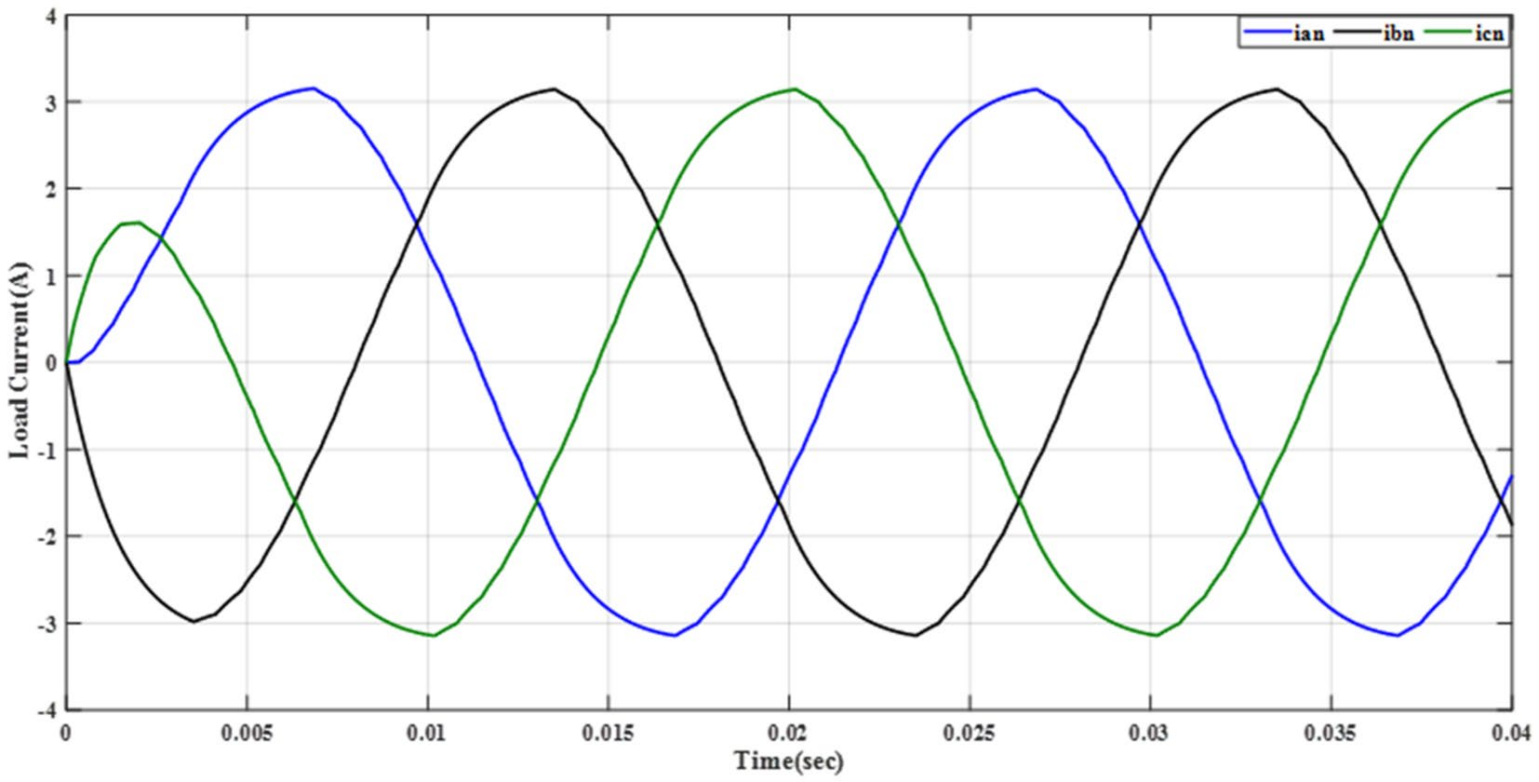
Line currents of RL load after implementing the GWO technique.

As demonstrated in Fig. 27, the line voltage experienced an increase in the number of voltage levels, as indicated by Eq. (7). This results in a waveform closely resembling a sine wave, resulting in reduced harmonics, as depicted in Fig. 28. In addition to optimizing the switching process, the improved line voltage can also contribute to its enhancement. Figure 28 illustrates the spectrum of line voltage, providing insights into the frequency components of the voltage across all three phases. In addition, Fig. 29 presents the three-phase current with a resistance of a value ${R}_{load}=10\Omega$ and inductance of a value ${L}_{load}=15\mathrm{ mH}$ at the same values of ${m}_{a}$ and ${m}_{f}$. It is obvious that it closely resembles a pure sine wave. This observation is supported by a low THD value of 1.5%. A low THD indicates that the current waveform is predominantly composed of the fundamental frequency, with minimal harmonic distortion. This information is valuable in evaluating the efficiency and performance of the three-phase electrical system under consideration.

**Figure 30:**
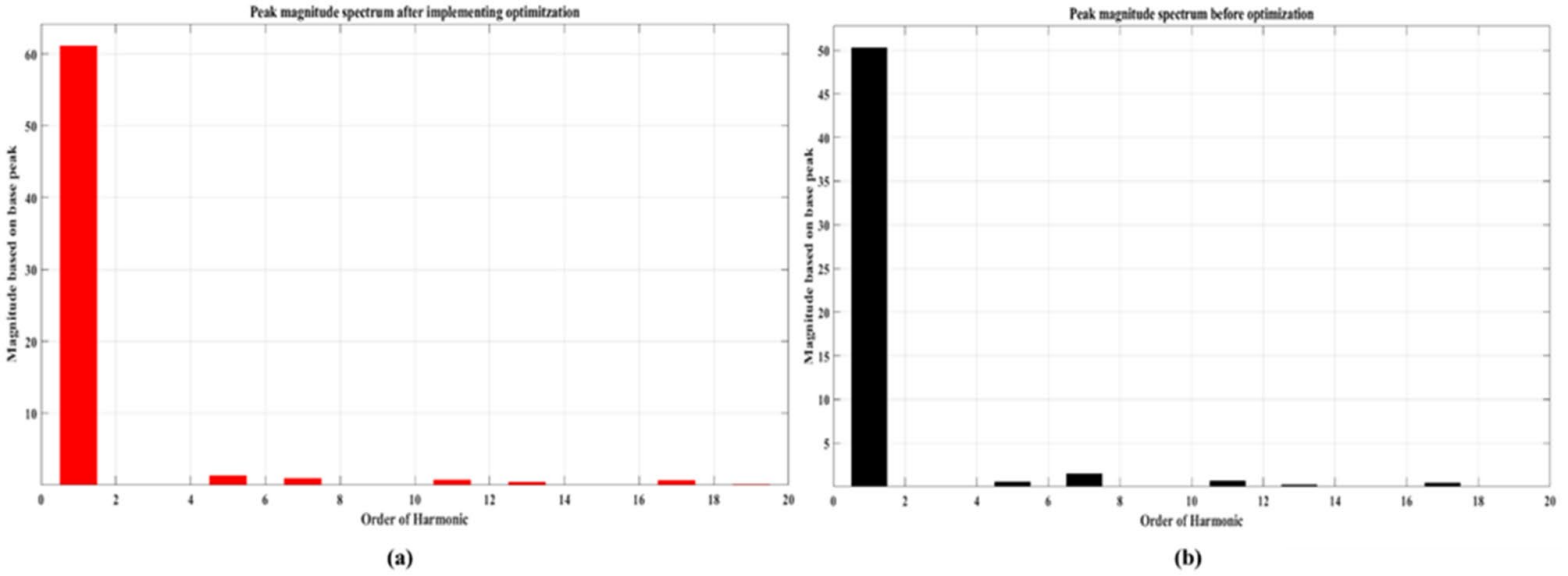
Comparison between low order harmonics, (**a**) before optimization (**b**) after optimization.

The impact of the optimization process on the individual low-order harmonics can be observed by analyzing Fig. 30. The 7th, 11th, and 17th harmonics were significantly reduced compared to the initial state. Additionally, the value of the fundamental component has increased from 50 to 60 V. These changes in the harmonic spectrum indicate that the optimization process has effectively shifted and improved the distribution of harmonics in the system. The optimization process has mitigated the presence of unwanted frequency components in the electrical signal. This reduction is beneficial as it helps to minimize distortion and improve the overall quality of the power signal. The increase in the value of the fundamental harmonic further contributes to enhancing the system performance, as the fundamental harmonic carries the essential information and desired functionality of the power signal.

### Comparison with the PSO and GA techniques

**Figure 31:**
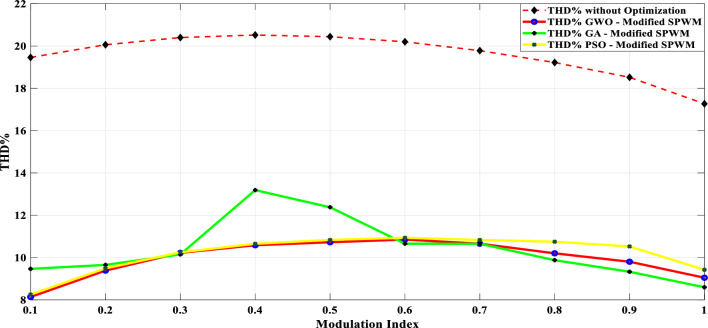
THD% of phase-voltage versus the modulation index.

**Figure 32:**
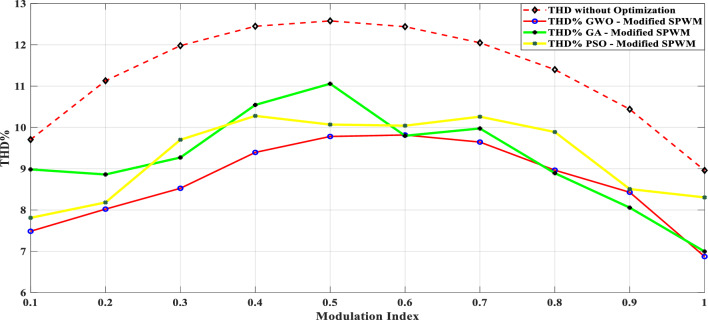
THD% of line-voltage versus the modulation index.

Table 8 presents a comprehensive comparison of the results obtained from the proposed modified SPWM scheme optimized using the GWO with the proposed SPWM scheme tuned with the GA, and PSO algorithms in terms of THD for phase voltage, line voltage, and load currents with modulation index variation. This table clearly shows that the proposed technique incorporating AI techniques is more robust and efficient than the proposed techniques without optimization. The THD was greatly reduced, allowing this topology to be used without the need for a filter, lowering the overall system cost. Figure 31 indicates the THD% of phase voltage versus the modulation index while Fig. 32 shows THD% of line voltage versus the modulation index. Table 8 demonstrates that the %THD in the 3-phase voltage is reduced to a value of 6.87201 instead of 6.99409 and 8.30455 using the GA and PSO techniques, respectively. It is concluded from Table 8 and Figs. 31 and 32 that the findings clearly demonstrated that the results implementing the proposed modified SPWM control scheme optimized using the GWO algorithm is better than those results obtained using the proposed modified SPWM control scheme tuned using the GA, and PSO technique. The proposed scheme significantly reduced the THD values for both phase and line voltages. Additionally, the GWO algorithm requires fewer parameters, making it an attractive choice for optimization tasks. While the GA algorithm yields better results compared to PSO, it falls slightly behind the GWO algorithm in terms of THD reduction.

**Table 8:** Efforts implementing the proposed scheme in comparison with the GA, and PSO technique.

Method	${m}_{a}$	Sector_1_	Sector_2_	Sector_3_	Sector_4_	Sector_5_	Sector_6_	$TH{D}_{v, ph}$	$TH{D}_{v, line}$	$TH{D}_{i,L}$
GWO	0.1	0.04827	0.14313	0.27297	0.37072	0.51721	0.67958	8.13747	7.48505	0.9888
	0.2	0.02843	0.14895	0.24599	0.38337	0.52231	0.69577	9.38548	8.02109	1.12657
	0.3	0.07453	0.14770	0.23988	0.34416	0.50525	0.66701	10.22370	8.5257	1.31917
	0.4	0.05733	0.14081	0.23280	0.37535	0.52540	0.66417	10.58343	9.39443	1.45787
	0.5	0.05813	0.14356	0.28291	0.35118	0.48279	0.64597	10.72515	9.78066	1.73621
	0.6	0.0508	0.15024	0.28134	0.38887	0.53276	0.64414	10.85153	9.81647	1.23572
	0.7	0.05080	0.15581	0.27726	0.36078	0.52262	0.64616	10.65534	9.64516	1.22551
	0.8	0.05540	0.15443	0.26357	0.36587	0.51653	0.68797	10.20113	8.96454	1.44671
	0.9	0.07115	0.14794	0.25821	0.36855	0.50735	0.62881	9.80255	8.42908	1.53238
	1	0.07626	0.14667	0.26431	0.35873	0.49071	0.67475	9.04738	**6.87201**	0.98657
GA	0.1	0.03364	0.20571	0.28641	0.37463	0.50903	0.61397	9.46602	8.98308	2.36093
	0.2	0.07614	0.15654	0.24507	0.40722	0.53645	0.70121	9.65298	8.86025	0.89539
	0.3	0.04224	0.18068	0.23961	0.38201	0.52281	0.64828	10.1495	9.27096	1.23981
	0.4	0.04287	0.16226	0.17969	0.31049	0.57870	0.66047	13.1891	10.54464	3.33606
	0.5	0.06535	0.17958	0.18569	0.36781	0.55926	0.60262	12.3781	11.05821	2.04748
	0.6	0.05061	0.14101	0.26183	0.40334	0.50108	0.64293	10.6561	9.79779	1.34021
	0.7	0.03539	0.15264	0.26982	0.40533	0.50365	0.65836	10.6541	9.97481	1.15497
	0.8	0.04447	0.14150	0.26336	0.38537	0.50676	0.66681	9.87649	8.89192	1.10066
	0.9	0.06128	0.14617	0.26364	0.38496	0.50631	0.66776	9.32887	8.05727	1.0318
	1	0.06134	0.14250	0.26432	0.38525	0.50691	0.66849	8.59569	**6.99409**	1.03085
PSO	0.1	0.05762	0.14958	0.26943	0.38223	0.51006	0.63877	8.25298	7.81054	1.39223
	0.2	0.05066	0.18333	0.25429	0.36184	0.53129	0.66923	9.49119	8.18291	1.11489
	0.3	0.05612	0.17666	0.25039	0.37994	0.48696	0.70501	10.24987	9.70097	1.23413
	0.4	0.02919	0.13801	0.27271	0.37551	0.51982	0.69104	10.65221	10.27922	0.99982
	0.5	0.01661	0.15838	0.25394	0.32273	0.44673	0.55068	10.83528	10.06921	3.81101
	0.6	0.03563	0.17298	0.20342	0.31806	0.40419	0.69261	10.93693	10.04216	4.27878
	0.7	0.05224	0.13625	0.21328	0.30297	0.52538	0.91361	10.83384	10.25981	5.71903
	0.8	0.00862	0.15347	0.20701	0.33011	0.46088	0.75321	10.74942	9.88894	3.69939
	0.9	0.06319	0.13281	0.24608	0.31634	0.53762	0.70336	10.52709	8.50726	2.28451
	1	0.02911	0.09992	0.27626	0.30164	0.43151	0.72215	9.42193	**8.30455**	3.77656

Significant values are in [bold].

The impact of the three applied techniques is shown in Figs. 31 and 32. These figures highlight the changes and differences resulting from implementing each technique. This visual representation provides a clearer understanding of the performance improvements achieved using the respective techniques. After implementing the GWO technique, the THD value was reduced to 6.87% measured at ${m}_{a}$ = 1. This value is lower than the THD values specified in the IEEE Standard 519^58^, which mandates a maximum THD of 8%. This result demonstrates the effectiveness of the GWO approach in achieving improved power quality by reducing harmonic distortion levels below the acceptable limits set by the IEEE Standards.

### Experimental validation

**Figure 33:**
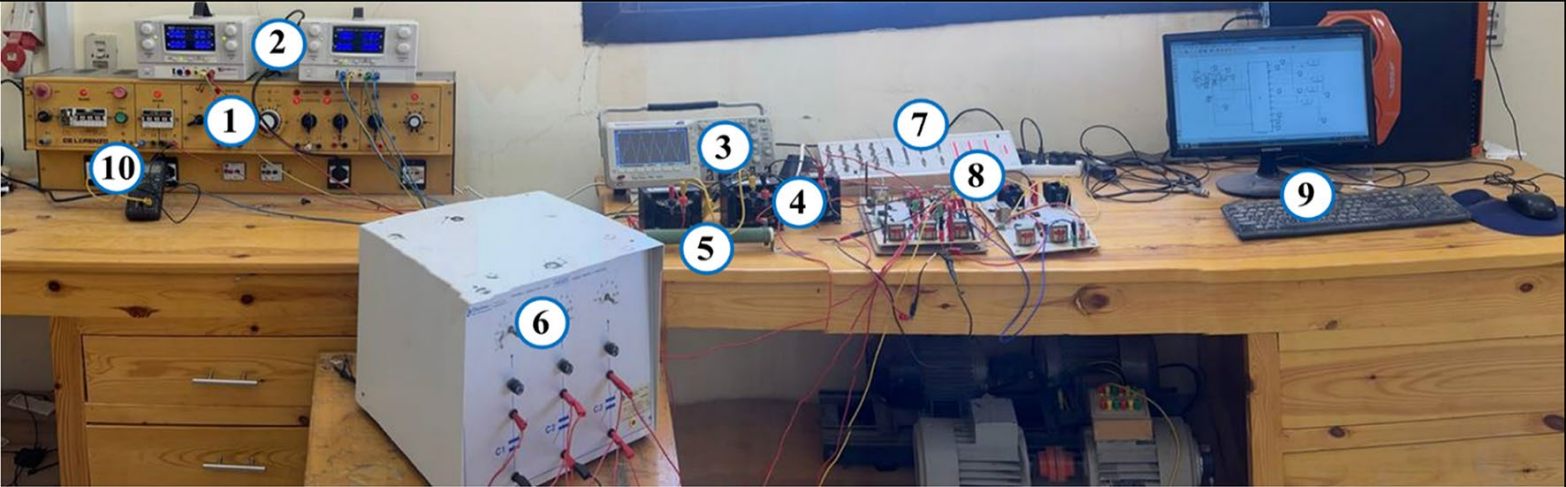
Experimental complete setup (1- DL1013M2 power supply module, 2- DC power supply, 3-Oscilloscope, 4- Inductive load, 5- Resistive load, 6- Capacitor bank, 7- DS1104 R&D Controller Board 8- Cascaded half-bridge MLI 9- PC with Simulink and hardware support and 10- multi-meter).

The performance assessment of the implemented fifteen-level inverter was conducted through utilizing a hardware prototype configuration. The fifteen-level inverter, which was employed in a single-phase system and powered by DC sources is represented in Fig. 33. Due to the capabilities of laboratory, the PV modules are replaced by the variable DC supply. The generation of the 15-level involves the utilization of only 10 switches, wherein each switch is a power MOSFET (IRFP460). The measurements are acquired using a Tektronix digital oscilloscope. The hardware setup employed the DS1104 R&D Controller Board^59^. Moreover, it has been compatibly implemented using MATLAB/Simulink® R2017b on a PC equipped with an Intel (R) Core i7, 2.7 GHz CPU. and subsequently compiled into a real-time system.

### Hardware setup descriptions

**Figure 34:**
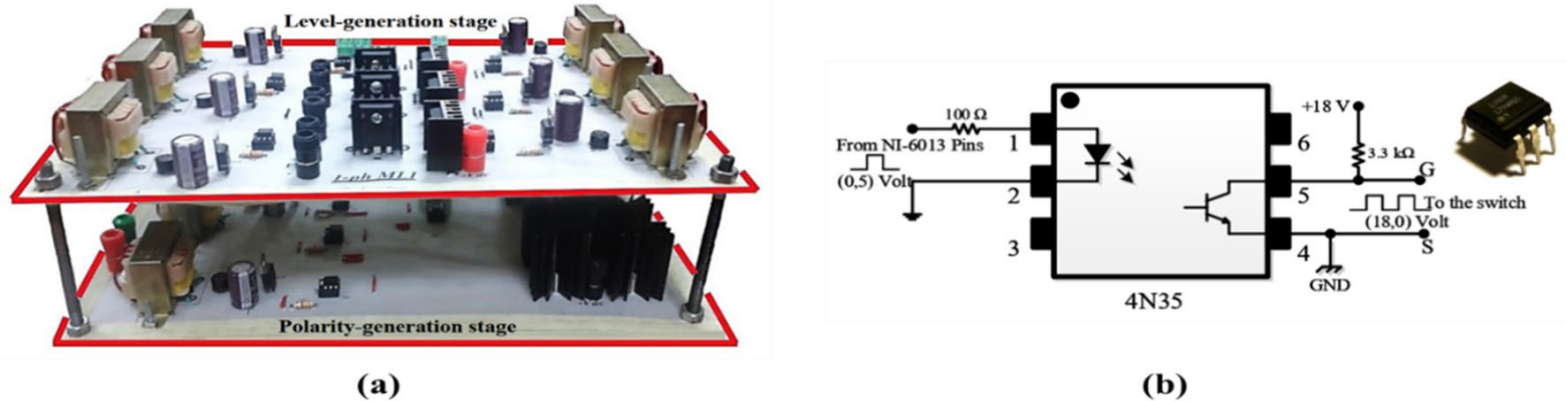
(**a**) Photograph of the manufactured prototype of the 15-level MLI, (**b**) Schematic diagram of gate-driver circuit.

An inverter circuit has been implemented using a controllable switch that incorporates a diode. This switch can conduct current and block voltage in both directions. It requires only one gate drive signal with an isolated power supply. Therefore, for the specific inverter design, only ten switches with ten gate-drives and ten isolated power supplies are needed, considering that there are two commonly sourced switches in the polarity-generation stage. The chosen switch for each of the ten positions is an IRFP460 (Intersil Corporation, Milpitas, CA, USA) (500 V–20 A) power MOSFET, A photograph of the actual inverter prototype is presented in Fig. 34a. Each MOSFET is mounted on a heat sink for cooling purposes, which reduces the MOSFET temperature during operation. The gate signals necessary for controlling the switches in the inverter circuits are connected to the controlled switches via gate-driver circuits. These circuits utilize opto-coupler devices, which are well-suited for the intended application. The output signal of the opto-coupler can be inverted or non-inverted depending on how the opto-coupler is connected. Figure 34b shows the schematic diagram of a single gate driver circuit. These gate-drive circuits are integrated into the same board as the inverter.

The DS1104 R&D Controller Board (dSPACE, Westphalia, Germany)^59^ is a powerful and versatile hardware module designed for real-time control. Its compact and robust controller offers high-performance computing capabilities and extensive I/O connectivity. It is equipped with a powerful dual-core processor (MPC8240 processor with PPC603e core, 250 MHz), ample memory (32 MB SDRAM), and a range of interfaces such as Ethernet, USB, and CAN. The CP1104 controller is specifically designed to work seamlessly with dSPACE software, enabling engineers to develop and test complex control algorithms easily. In the experimental setup, a static load was employed with a resistance value of 10Ω. Three coils were incorporated with one coil having an inductance value of 5 mH. By connecting these three coils in series, the total inductance becomes 15 mH, enabling a power factor of 0.9. To obtain a capacitive load with a power factor of approximately 0.9 leading, a capacitor bank was utilized. The capacitance consisted of 680 $\upmu$F, combined with a resistance value of 10 ohms. This configuration allowed for the desired power factor to be realized, facilitating the evaluation and analysis of the proposed scheme’s effectiveness under different load conditions.

### Experimental results

**Figure 35:**
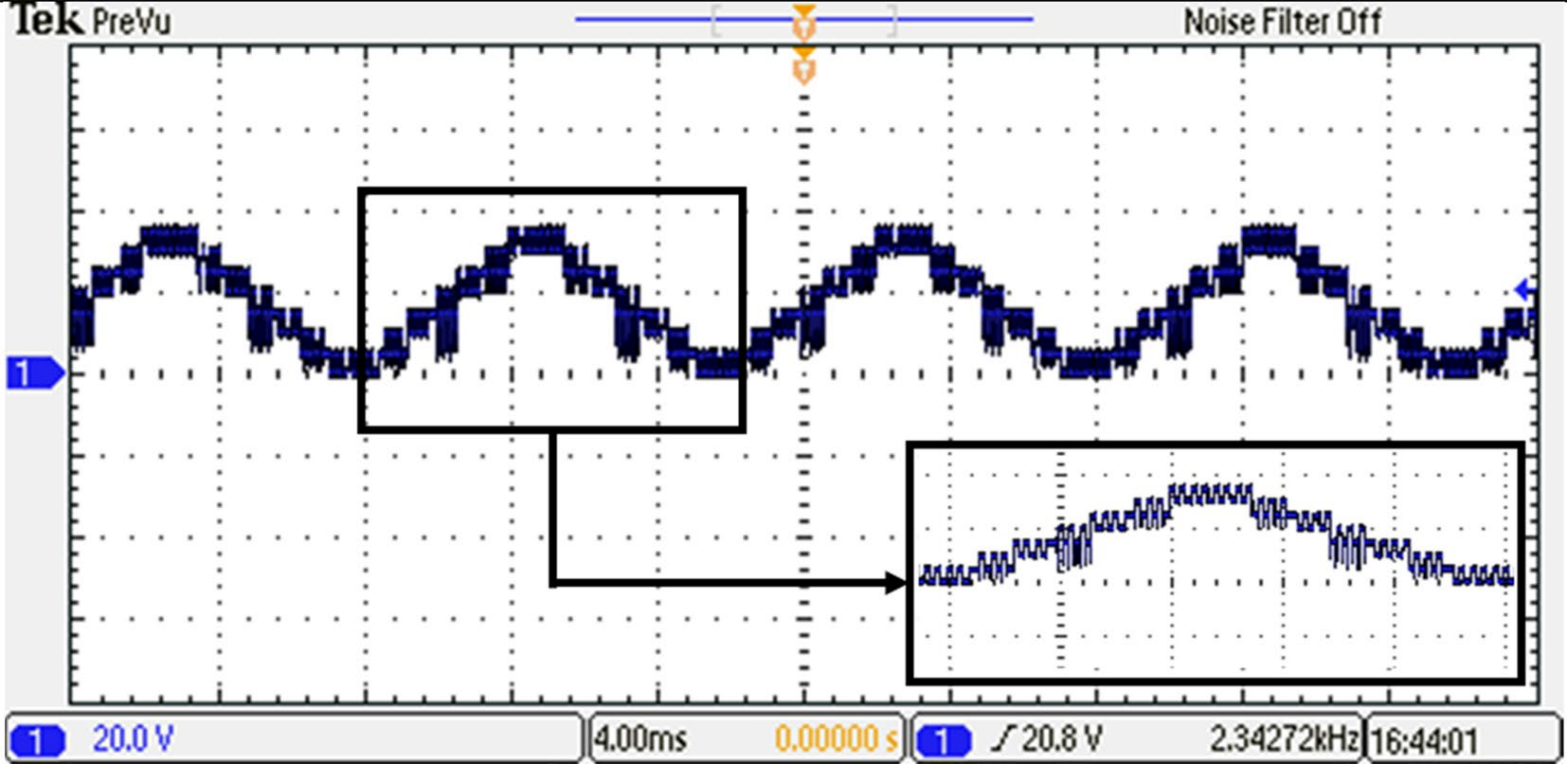
The inverter’s DC link voltage before optimization.

In this experimental study, the performance of a 15-level inverter was investigated. The experimental setup included two stages: the first stage generated a stepped DC voltage, as shown in Fig. 35. This stepped DC voltage was then converted into an AC voltage by the second stage of the inverter. Based on the required switching angles that are determined by the modified SPWM method with equal step offline ($Sector=\frac{1}{7}$), the DS1104 R&D Controller Board produced the required pulses in a single phase for all switches. A carrier frequency of ${f}_{c}$= 10 kHz is used.

**Figure 36:**
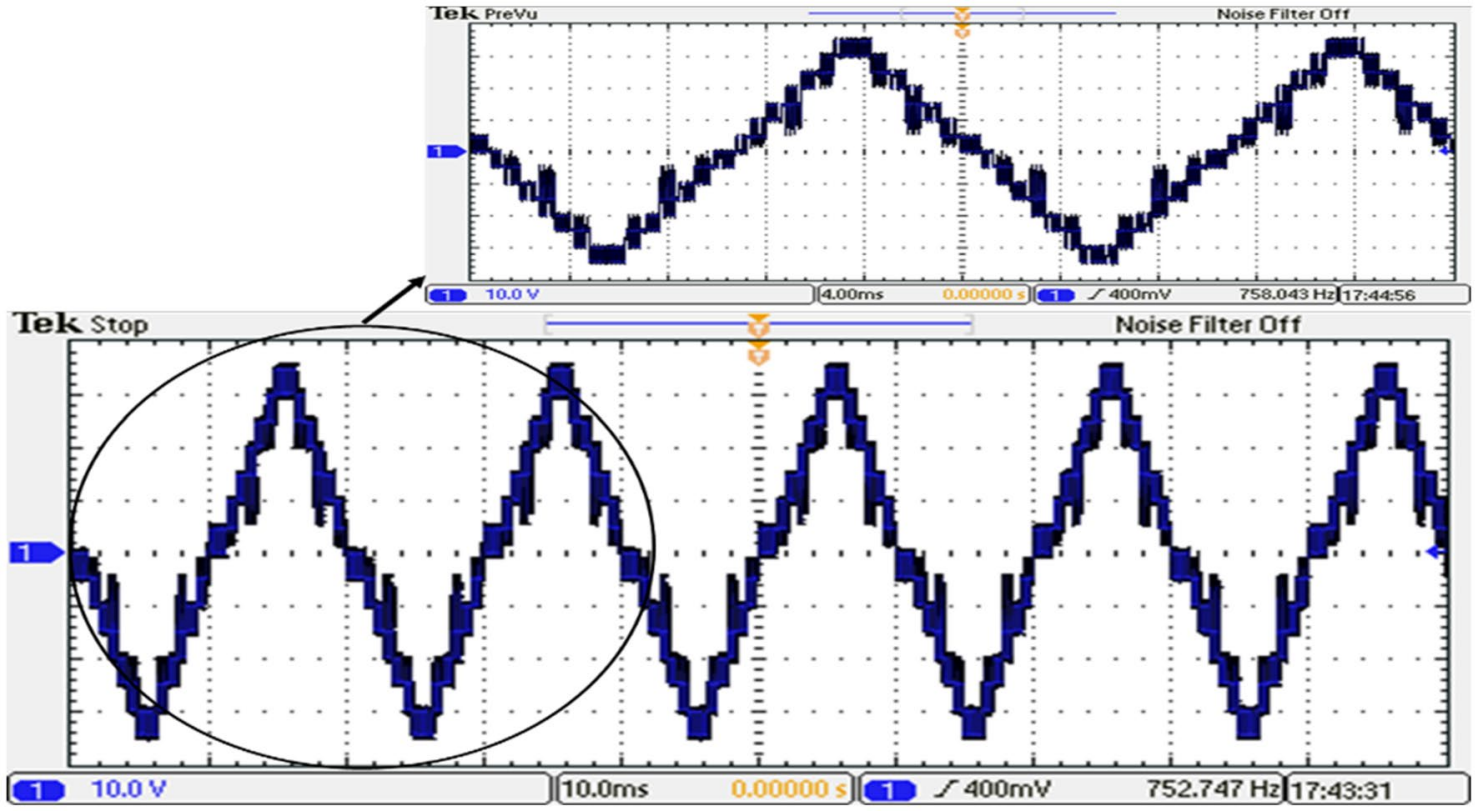
Output voltage of the inverter before optimization.

**Figure 37:**
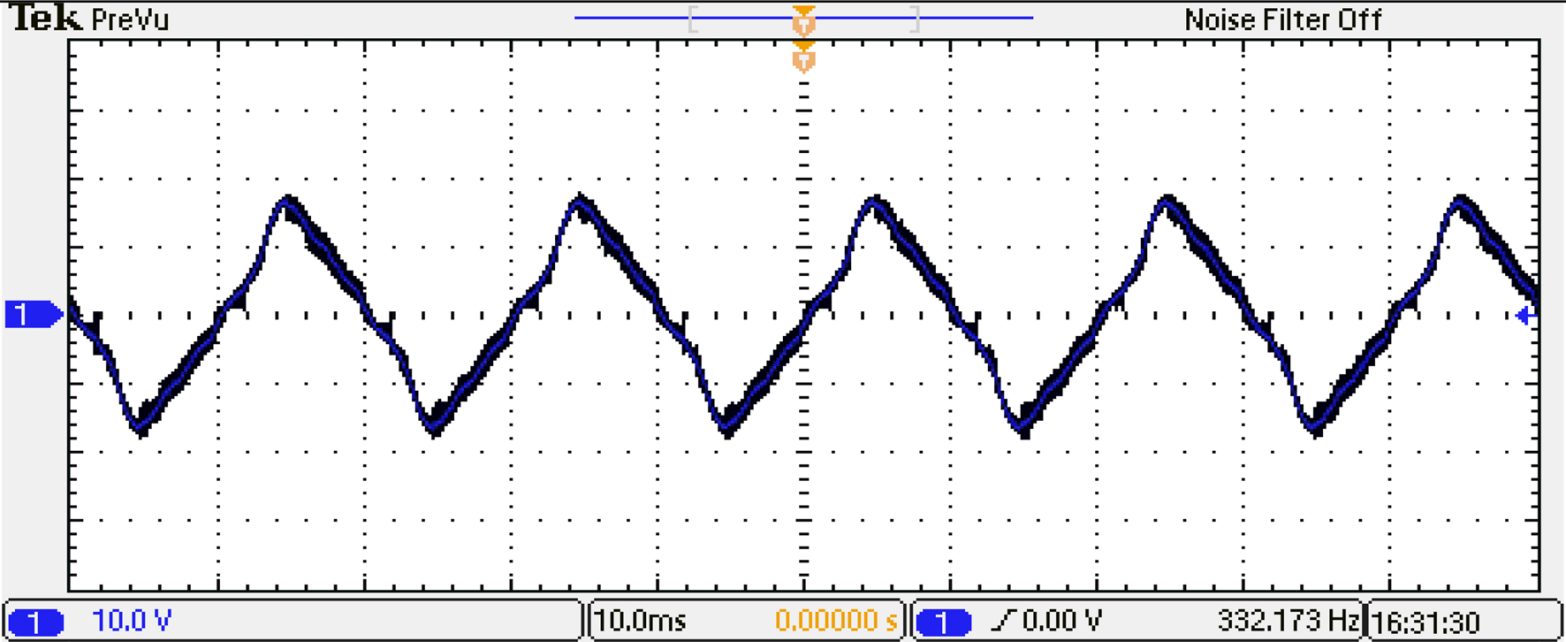
Load current of the inverter for RL load before optimization.

To analyze the output of the inverter, the phase-output voltages are examined. Figure 36 indicates the phase-output voltages of the 15-level inverter without optimization technique at ${m}_{a}=0.9$ and ${m}_{f}=100$, operating at a frequency of ${f}_{m}$ = 50 Hz. highlighting the distinct voltage levels achieved by the system. Furthermore, Fig. 37 displays the load current, providing a comprehensive view of the AC output of the inverter.

**Figure 38:**
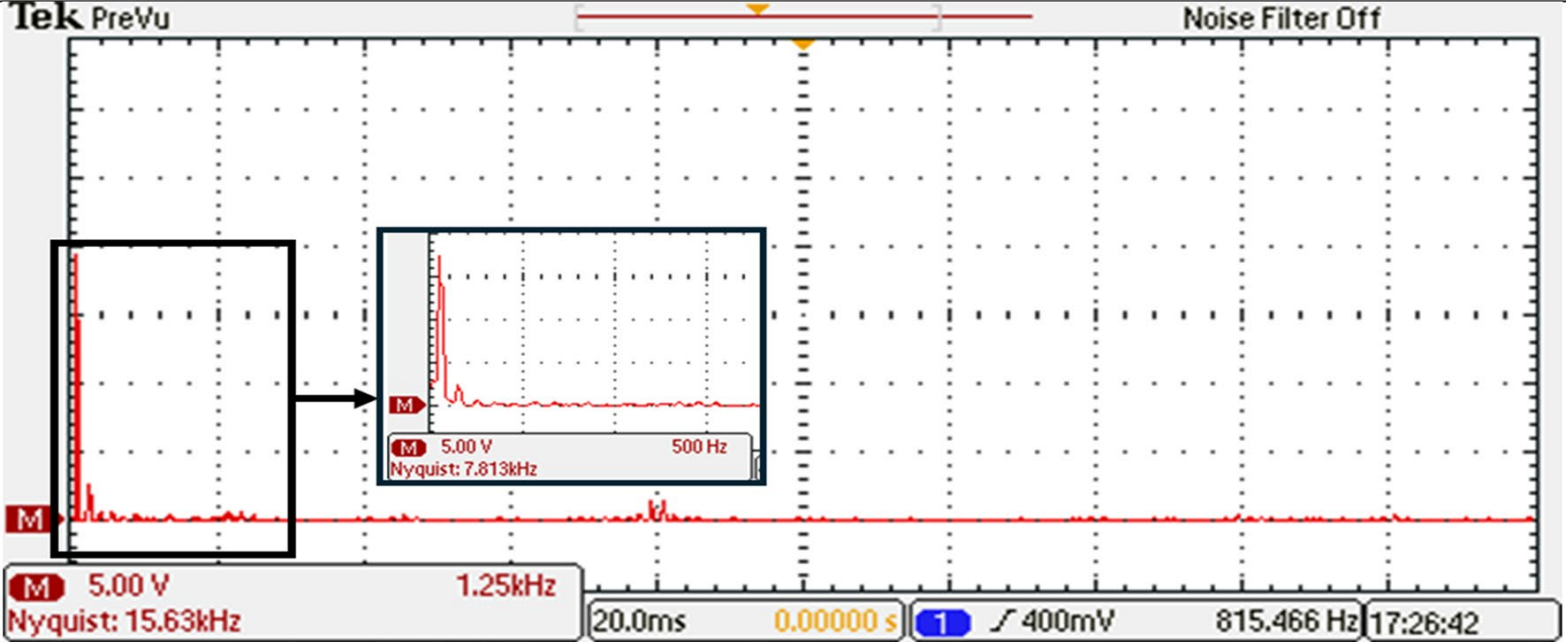
Resultant spectrum of the output voltage inverter before optimization.

To gain further insights into the performance of the 15-level inverter, FFT analysis on the phase voltage is conducted. The FFT analysis, presented in Fig. 38, allowed to analyze the frequency components present in the output waveform. The harmonic content and the overall quality of the phase voltage produced by the 15-level inverter are assessed by examining the FFT analysis. The harmonic content and the overall quality of the phase voltage produced by the 15-level inverter are assessed by examining the FFT analysis.

**Figure 39:**
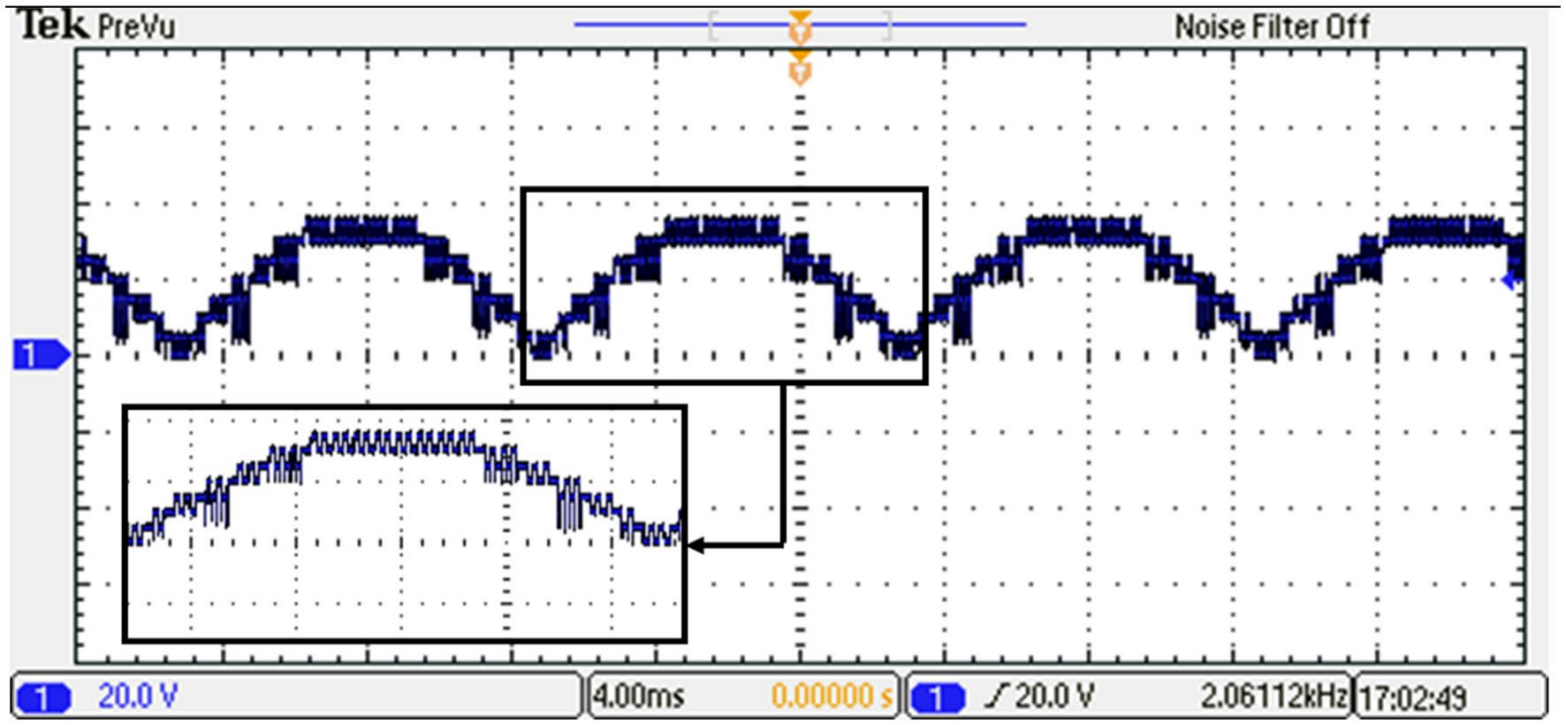
The inverter’s DC link voltage with GWO technique.

**Figure 40:**
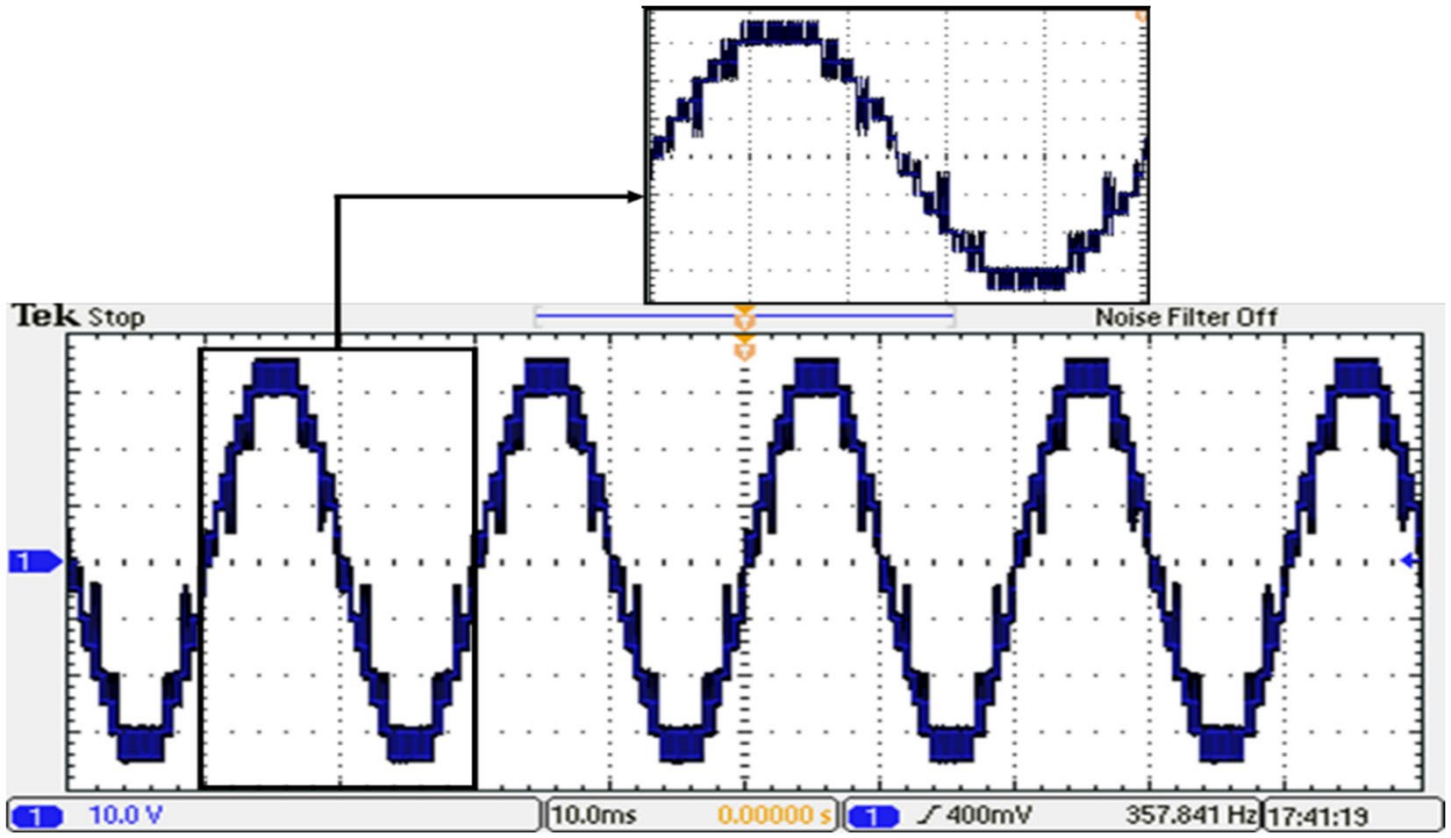
Output voltage of the inverter with GWO technique.

**Figure 41:**
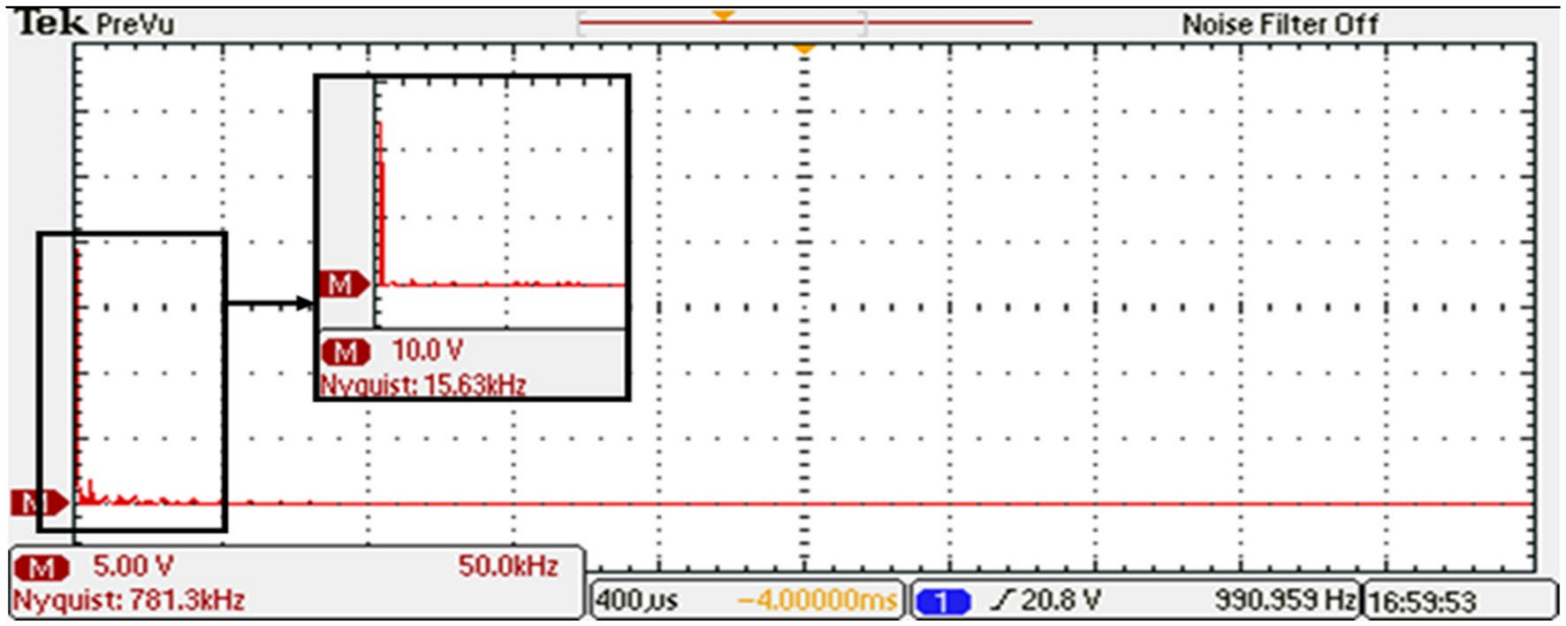
Resultant spectrum of the output voltage inverter with GWO technique.

**Figure 42:**
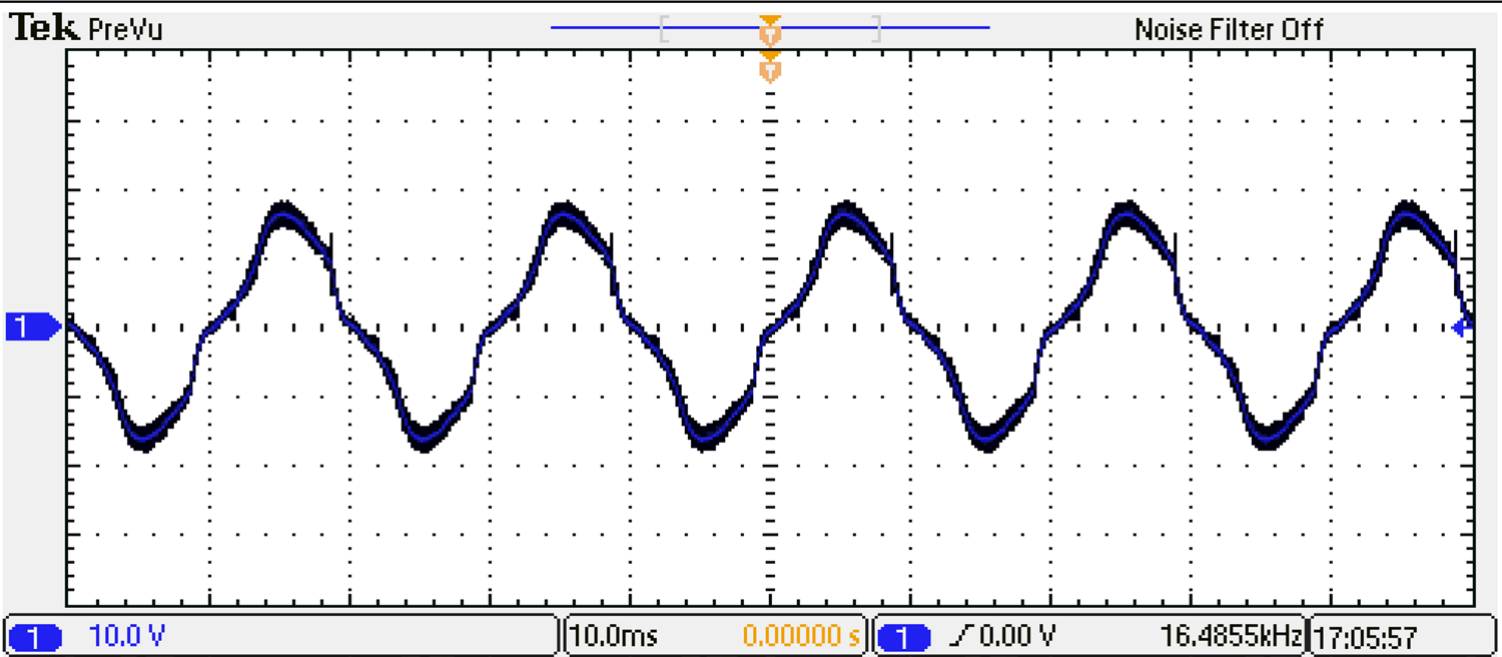
Load current of the inverter for RL load with GWO technique.

Based on the required switching angles that are determined by the proposed control scheme optimized using the GWO and its efforts presented in Table 7, the DS1104 R&D Controller Board produced the required pulses in single phase for all switches. The resulting DC link voltage of the inverter and output voltage waveforms after implementing the GWO technique at ${m}_{a}=0.9$ and ${m}_{f}=100$ are depicted in Figs. 39 and 40, while Fig. 41 presents the spectrum of the output voltage under the same modulation index where the spectrum has been improved. Additionally, the waveforms of the load currents are displayed in Fig. 42 for power factors of 0.9 lagging, respectively. These waveforms clearly indicate a more sinusoidal characteristic of the load currents.

To match the laboratory’s supply capabilities, the input voltages of the inverter were adjusted to 5 V, 10 V, and 20 V, resulting in an approximate root mean square value of the output voltage (${V}_{rms}$) with a value of 21 V. The experimental setup yielded the following results which were optimized by the GWO technique as indicated in the subsection “Implementation of the GWO technique”.

Notches or distortions in the output voltage waveform of MLI in Fig. 40 can be caused by several factors. These include voltage balancing issues, which occur when there is an imbalance between the different voltage levels. Switching frequencies can also contribute to notches and distortions, especially under certain load conditions. Dead time, which is introduced to prevent simultaneous conduction of power devices, can create notches in the waveform. Additionally, nonlinear load impedance, such as certain power electronics or motor drives, can affect the MLI’s behavior and lead to waveform variations.

**Figure 43:**
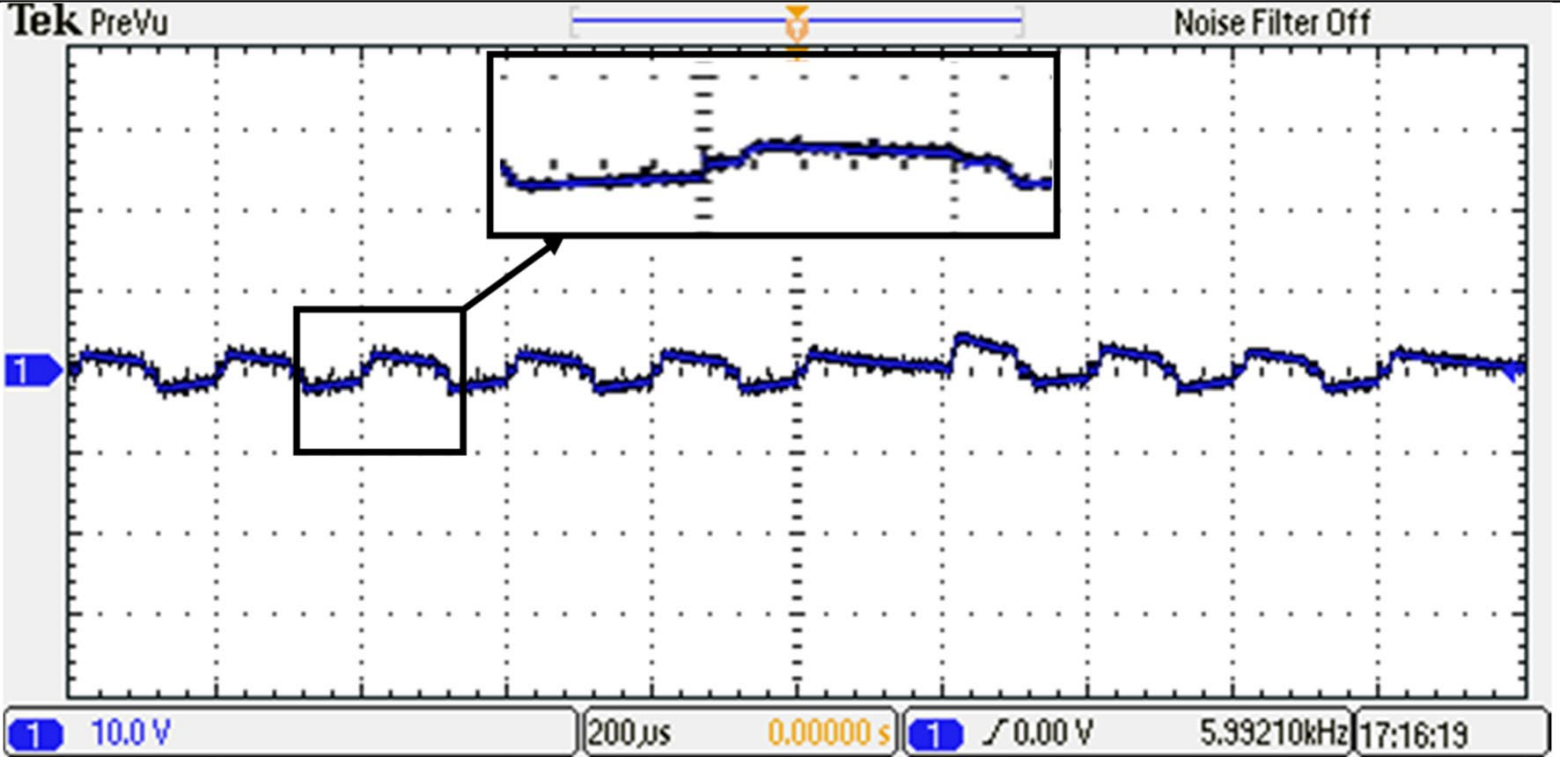
Load current of the inverter for RC load with GWO technique.

To validate the effectiveness of the proposed scheme, a change was made from an inductive load to a capacitive load with a power factor of 0.9 leading. This alteration was implemented to assess the performance and efficiency of the system under different load conditions. The load current for the capacitive load, ${R}_{load}=10\Omega$, a capacitance per phase of ${C}_{load}=680\mathrm{ \mu F}$, is shown in Fig. 43.

Overcoming the challenges of firing high-side switches in H-bridge inverter configurations can be difficult. One widely used approach to address these challenges and effectively drive both low-side and high-side MOSFETs is through a technique known as bootstrapping, which is discussed in^60^.

**Figure 44:**
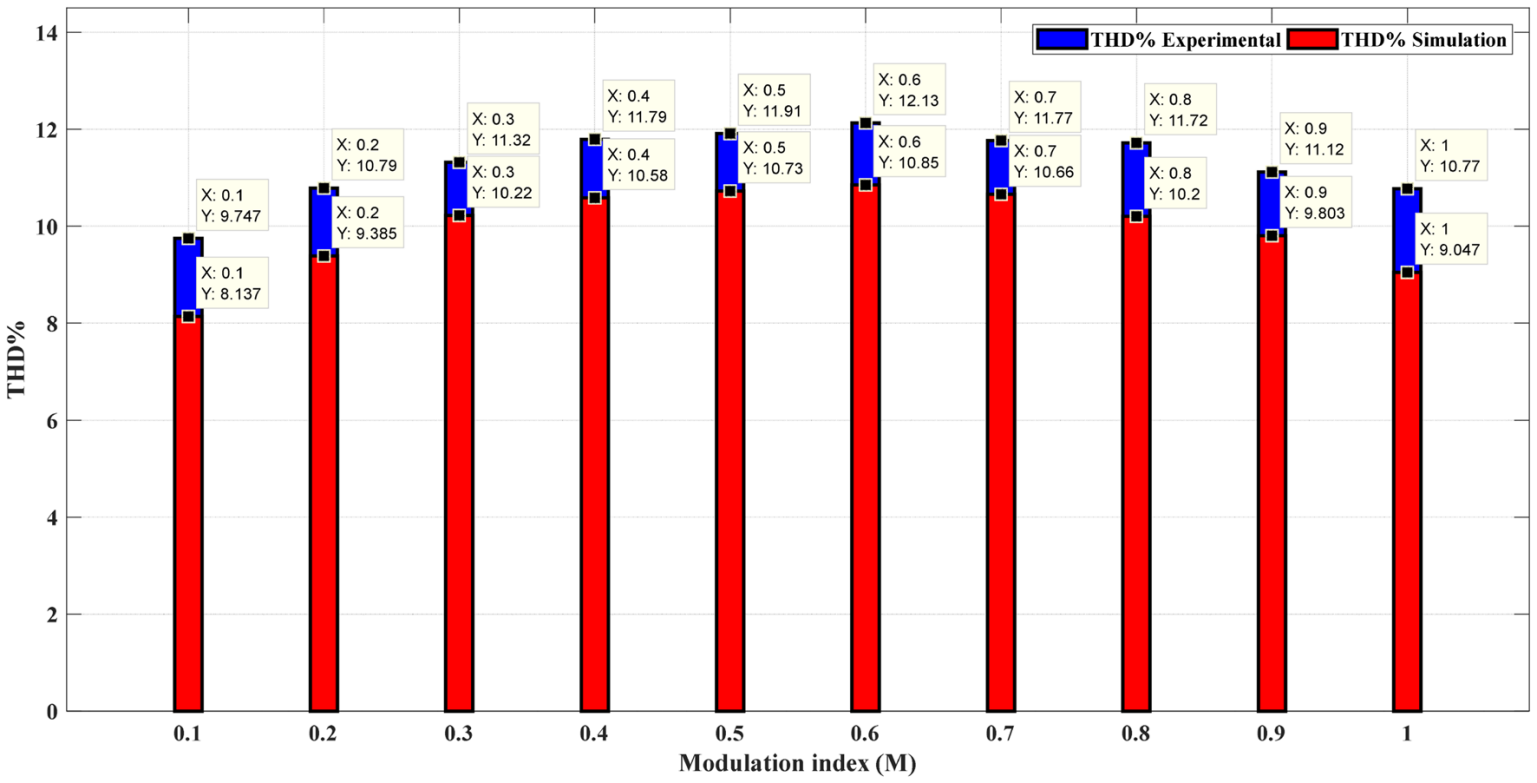
Comparison between the simulation and experimental results for single-phase 15-level MLI for GWO.

It is concluded, from the experimental results, that the experimental validation of the simulation results for the modeled system was successfully conducted as shown in Fig. 44. The prototype can generate the 15-level voltage with the optimized switching sectors and without optimization (Equally stepped method). It verified the effectiveness, accuracy, and robustness of the proposed modified SPWM control scheme and hence the implemented fifteen-level inverter.

## Conclusion

A novel modified SPWM control scheme for a three-phase half-bridge cascaded MLI powered by PVs has been proposed in this article. A reduced number of switching components with reliability and simplicity of implementation characterized the introduced MLI configuration. The proposed modified SPWM implemented only three signals which simplified and facilitated its control and implementation. GWO technique has been implemented to find the optimal switching angles of a three-phase 15-level CHB-MLI using the THD fitness function. For comparison, the proposed GWO-based modified SPWM control scheme has been compared with the modified SPWM and the modified SPWM optimized using the PSO and GA techniques. It is concluded that the findings demonstrated that the results implementing the proposed modified SPWM control scheme optimized using the GWO algorithm are better than those results obtained using the modified SPWM control scheme tuned using the GA, and PSO techniques. Simulation results verified that the THD was reduced by 50%. The results demonstrated the effectiveness of the GWO technique in achieving the desired THD reduction without the need for a filter, resulting in a THD value of 6.8% for the line voltage. The load current also became sinusoidal with a THD value of less than 1%, indicating a successful reduction in distortion. The proposed scheme significantly reduced the THD values for both phase and line voltages. Additionally, the GWO algorithm requires fewer parameters, making it an attractive choice for optimization tasks. Furthermore, the paper conducted experimental validation by building a laboratory prototype of the proposed system. The experimental results validated the proposed scheme and provided strong evidence for the effectiveness of the proposed scheme. The proposed method can be extended to control higher levels of MLIs.

### Supplementary Information

Supplementary Information 1.

## Data Availability

The data that support the findings of this study are available from the corresponding author upon reasonable request.

## Abbreviations

DE: Differential evolution
${\text{FFT}}$: Fast Fourier transform
GA: Genetic algorithm
GWO: Grey wolf optimization
HSFM: High-frequency switching modulation
LSF: Low switching frequency
MIs: Modulation indices
MLIs: Multilevel inverters
MMC: Modular multilevel converter
PSO: Particle swarm optimization
RESs: Renewable energy sources
RKO: Runge Kutta optimization
SHE-PWM: Selective harmonic elimination-based PWM
TLO: Teaching–learning-based optimization
UPFC: Unified power flow control

## List of symbols

${f}_{c}$: Carrier signal frequency
${f}_{m}$: Modulating signal frequency
$m$: Number of levels for phase voltage
$${m}_{a}$$: Modulation index
$${m}_{f}$$: Modulation frequency
$n$: Number of cascaded fundamental units
${m}_{line}$: Number of levels for line voltage
$TH{D}_{i,L}$: Percentage of THD for load current
$TH{D}_{v, line}$: Percentage of THD for line-voltage
$${V}_{o}$$: Output voltage
${v}_{{o}_{1}}$: DC output voltage for fundamental unit
