# Supplementary material for: A grey wolf optimization-based modified SPWM control scheme for a three-phase half bridge cascaded multilevel inverter
Source: Sci Rep. 2024 Mar 25;14:7016. doi: 10.1038/s41598-024-57262-0 (PMC10963797; doi:10.1038/s41598-024-57262-0)
Supplement: Supplementary file 1 — Supplementary Information 1. [file 41598_2024_57262_MOESM1_ESM.pdf]

Appendix A: PV parameters.

**Parameters**	**Values**
Maximum output power (-0/+5)	125 W
Voltage at maximum output power	124 V
Open circuit voltage ($V_{o.c}$)	165 V
Short circuit current ($I_{s.c}$)	1.17 A
Temperature coefficient of $V_{o.c}$	-0.4 V/°C
Temperature coefficient of $I_{s.c}$	+0.07 A/°C
Temperature coefficient of $P_{o,max}$	-0.3 W/°C
Boltzmann's constant	1.3805×10^{-23} J/K
Electric charge	1.6×10^{-19} coulombs

Appendix B: Main program of modified SPWM technique.

```
%Code of modified SPWM for phase A
%for phase b and c, state value will change to
achieve the balance three phase voltage.
function [s1, s2, s3, s4, s5, s6]= fcn(u,v,z)
persistent state;
if isempty(state)
 state=0;
end
persistent S1;
if isempty(S1)
 S1=0;
end
persistent S2;
if isempty(S2)
 S2=0;
end
persistent S3;
if isempty(S3)
 S3=0;
end
persistent S4;
if isempty(S4)
 S4=0;
end
persistent S5;
if isempty(S5)
 S5=0;
end
persistent S6;
if isempty(S6)
 S6=0;
end
switch (state)
    case 0
        if (v>=z)
        S1=1;S2=0;S3=0;S4=1;S5=0;S6=1;
        else
        S1=0;S2=1;S3=0;S4=1;S5=0;S6=1;
        end
        if(u>1/7)
          state=1;
        end
       case 1
            if (v>=z)
        S1=0; S2=1;S3=1;S4=0;S5=0;S6=1;
        else
        S1=1;S2=0;S3=0;S4=1;S5=0;S6=1;
            end
        if(u> 2/7)
          state=2;
        end
          case 2
               if (v>=z)
         S1=1;S2=0;S3=1;S4=0; S5=0;S6=1;
        else
         S1=0;S2=1;S3=1;S4=0;S5=0;S6=1;
               end
        if(u>3/7)
          state=3;
        end
           case 3
           if (v>=z)
         S1=0;S2=1;S3=0;S4=1;S5=1;S6=0;
           else
         S1=1;S2=0;S3=1;S4=0;S5=0;S6=1;
           end
        if(u>4/7)
          state=4;
        end
            case 4
        if (v>=z)
        S1=1;S2=0;S3=0;S4=1;S5=1;S6=0;
         else
       S1=0;S2=1;S3=0;S4=1;S5=1;S6=0;
         end
         if(u> 5/7)
          state=5;
         end
                 case 5
              if (v>=z)
              S1=0;S2=1;S3=1;S4=0;S5=1;S6=0 ;
              else
                 S1=1;S2=0;S3=0;S4=1;S5=1;S6=0;
              end
             if(u >6/7)
               state=6;
             end
               case 6
              if (v>=z)
            S1=1;S2=0;S3=1;S4=0;S5=1;S6=0;
             else
           S1=0;S2=1;S3=1;S4=0;S5=1;S6=0;
              end
             if(u<6/7)
              state=7
             end
              case 7
                  if (v>=z)
             S1=0;S2=1;S3=1;S4=0;S5=1;S6=0;
             else
                  S1=1; S2=0;S3=0;S4=1;S5=1;S6=0;
                  end
              if(u<5/7)
              state=8
              end
                   case 8
                  if (v>=z)
                 S1=1; S2=0;S3=0;S4=1;S5=1;S6=0;
             else
                  S1=0;S2=1;S3=0;S4=1;S5=1;S6=0;
                  end
                    if(u<4/7)
              state=9
                    end
        case 9
               if (v>=z)
                    S1=0;S2=1;S3=0;S4=1;S5=1;S6=0;
               else
                  S1=1;S2=0;S3=1;S4=0;S5=0;S6=1;
               end
                      if(u<3/7)
              state=10
                      end
                     case 10
               if (v>=z)
                   S1=1;S2=0;S3=1;S4=0;S5=0;S6=1;
               else
                  S1=0;S2=1;S3=1;S4=0;S5=0;S6=1;
               end
                      if(u<2/7)
              state=11
                      end
              case 11
               if (v>=z)
                 S1=0;S2=1;S3=1;S4=0;S5=0;S6=1;
               else
                  S1=1;S2=0;S3=0;S4=1;S5=0;S6=1;
               end
                       if(u<1/7)
              state=12
                 end
                   case 12
               if (v>=z)
                  S1=1;S2=0;S3=0;S4=1;S5=0;S6=1;
               end
                       if(u>0)
              state=0
                       end
end
s1=S1; s2=S2;s3=S3;s4=S4;s5=S5;s6=S6;
```

Appendix C: Main program for the implemented GWO.

```
%GWO MATLAB code for determining optimal switching angles for 15-level MLI
global x1 x2 x3 x4 x5 x6
if all([0 < x1, x1 < x2, x2 < x3, x3 < x4, x4 < x5, x5 < x6, x6 < 1])
    x1 = [];x2 = [];x3 = [];x4 = [];x5 = [];x6 = [];
    % GWO parameters
    SearchAgents_no = 50; % Number of search agents
    Max_iteration = 50; % Maximum number of iterations
    lb = [0 0 0 0 0 0];
    ub = [1 1 1 1 1 1];
    % Initialization
    Positions = initialization(SearchAgents_no, ub, lb);
    % Variable to store fitness values of each iteration
    fitness_curve = zeros(Max_iteration, 1);
    for iter = 1:Max_iteration
        % Evaluate fitness for each search agent
        fitness = zeros(SearchAgents_no, 1);
        for i = 1:SearchAgents_no
            fitness(i) = Fitness(Positions(i, :));
        end
        % Sort positions and fitness values
        [fitness, sortedIndices] = sort(fitness);
        Positions = Positions(sortedIndices, :);
        % Update best score and position
        Best_score = fitness(1);
        Best_pos = Positions(1, :);
        % Store the best fitness value of each iteration
        fitness_curve(iter) = Best_score;
        % Update the positions of search agents
        a = 2 - iter * (2 / Max_iteration); % a decreases linearly from 2 to 0
        for i = 1:SearchAgents_no
            for j = 1:size(Positions, 2)
                r1 = rand(); % random number between 0 and 1
                r2 = rand();
                A1 = 2 * a * r1 - a; % parameter A1
                C1 = 2 * r2; % parameter C1
                D_alpha = abs(C1 * Best_pos(j) - Positions(i, j)); % distance to alpha
                X1 = Best_pos(j) - A1 * D_alpha; % new position based on alpha
                r1 = rand();
                r2 = rand();
                A2 = 2 * a * r1 - a; % parameter A2
                C2 = 2 * r2; % parameter C2
                D_beta = abs(C2 * Best_pos(j) - Positions(i, j)); % distance to beta
                X2 = Best_pos(j) - A2 * D_beta; % new position based on beta
                r1 = rand();
                r2 = rand();
                A3 = 2 * a * r1 - a; % parameter A3
                C3 = 2 * r2; % parameter C3
                D_delta = abs(C3 * Best_pos(j) - Positions(i, j)); % distance to delta
                X3 = Best_pos(j) - A3 * D_delta; % new position based on delta
                Positions(i, j) = (X1 + X2 + X3) / 3; % update position
            end
        end
    end
    % Select the best individual from the final positions
    bestIndividual = Best_pos;
    final_answer = bestIndividual;
    x11 = final_answer(1);x22 = final_answer(2);x33 = final_answer(3);
    x44 = final_answer(4);x55 = final_answer(5); x66 = final_answer(6);
else
    error("The condition 0 < x1 < x2 < x3 < 1 is not satisfied.");
end
```
